# Albumin Kinetics in Patients Undergoing Major Abdominal Surgery

**DOI:** 10.1371/journal.pone.0136371

**Published:** 2015-08-27

**Authors:** Åke Norberg, Olav Rooyackers, Ralf Segersvärd, Jan Wernerman

**Affiliations:** 1 Department of Anaesthesia and Intensive Care, Karolinska University Hospital Huddinge, Stockholm, Sweden; 2 Department of Clinical Science, Intervention and Technology (CLINTEC), Karolinska Institutet, Stockholm, Sweden; 3 Division of Surgery, Department of Clinical Science, Intervention and Technology (CLINTEC), Karolinska Institutet at Karolinska University Hospital Huddinge, Stockholm, Sweden; Erasmus Medical Centre, NETHERLANDS

## Abstract

**Background:**

The drop in plasma albumin concentration following surgical trauma is well known, but the temporal pattern of the detailed mechanisms behind are less well described. The aim of this explorative study was to assess changes in albumin synthesis and transcapillary escape rate (TER) following major surgical trauma, at the time of peak elevations in two well-recognized markers of inflammation.

**Methods:**

This was a clinical trial of radiolabeled human serum albumin for the study of TER and plasma volume. Ten patients were studied immediately preoperatively and on the 2nd postoperative day after major pancreatic surgery. Albumin synthesis rate was measured by the flooding dose technique employing incorporation of isotopically labelled phenylalanine.

**Results:**

Fractional synthesis rate of albumin increased from 11.7 (95% CI: 8.9, 14.5) to 15.0 (11.7, 18.4) %/day (p = 0.027), whereas the corresponding absolute synthesis rate was unchanged, 175 (138, 212) versus 150 (107, 192) mg/kg/day (p = 0.21). TER was unchanged, 4.9 (3.1, 6.8) %/hour versus 5.5 (3.9, 7.2) (p = 0.63). Plasma volume was unchanged but plasma albumin decreased from 33.5 (30.9, 36.2) to 22.1 (19.8, 24.3) g/L. (p<0.001).

**Conclusion:**

Two days after major abdominal surgery, at the time-point when two biomarkers of generalised inflammation were at their peak and the plasma albumin concentration had decreased by 33%, we were unable to show any difference in the absolute synthesis rate of albumin, TER and plasma volume as compared with values obtained immediately pre-operatively. This suggests that capillary leakage, if elevated postoperatively, had ceased at that time-point. The temporal relations between albumin kinetics, capillary leakage and generalised inflammation need to be further explored.

**Trial Registration:**

clinicaltrialsregister.eu: EudraCT 2010-08529-21 ClinicalTrials.gov NCT01194492

## Introduction

Intravenous fluids are a cornerstone of the management of hypovolemia in connection with surgery, but the choice of fluid and the amount to be given is subject to a vigorous debate [1–4]. Following the suggested harm of starch in intensive care patients [5, 6], and the absence of a benefit when used perioperatively [7, 8], there is an increasing interest in albumin for intravenous fluid treatment when a colloid is considered. Although the preservation of colloid osmotic pressure during surgery by albumin reduced intestinal oedema [9], albumin infusions after the end of surgery have failed to show any benefit for the patients [10, 11]. Therefore, it remains unclear if albumin has a place in fluid treatment in the operating room.

Systemic inflammatory response syndrome (SIRS) is a response to a generalised inflammation that can be elicited by surgical trauma. Among the features of SIRS is an increased capillary leakage to albumin [12], contributing to a decrease in plasma albumin concentration (P-Alb). The physiological importance of low P-Alb is unclear, but a value below 25 g/L on the first postoperative day is a predictor of pancreatic fistula and major complications after pancreaticduodenectomy [13]. However, the time course of the capillary leakage in conjunction with surgery is not well characterised, but could be synchronous with the level of inflammation. A biomarker of generalised inflammation, plasma C-reactive protein, reaches its peak on the first to third post-operative day after major abdominal surgery. A peak in albumin leakage may hypothetically occur at the same time. How the synthesis of albumin is affected by surgery and inflammation is sparsely studied and results are variable depending on the timing in relation to the surgical procedure [14–17]. A recent review summarises these findings and concludes that an intraoperative decrease is followed by an increase in synthesis rate 8–10 days postoperatively [18]. A more thorough knowledge of albumin kinetics will contribute to a better design of future clinical studies to assess the role and timing for albumin treatment in modern fluid therapy perioperatively.

The aim of this explorative study was to describe changes in albumin kinetics caused by major surgical trauma by comparing measurements immediately before the surgical procedure with measurements on the 2^nd^ postoperative day. We hypothesized that both TER and absolute synthesis rate should increase. Stable isotopes were used to determine fractional synthesis rate (FSR) of albumin, and iodinated human serum albumin to assess TER and plasma volume.

## Materials and Methods

The study protocol for this trial and supporting TREND checklist are available as supporting information (S1 Protocol, and S1 TREND Checklist).This prospective open explorative hypothesis generating pilot study on albumin kinetic parameters in surgical patients was approved by the Ethical Review Board in Stockholm, the Radiation Safety Committee at Karolinska University Hospital Huddinge Sweden, and the Swedish Medical Product Agency (EudraCT 2010-08529-21). Furthermore, it was registered at www.clinicaltrials.gov trial number NCT01194492. The study was conducted according to Good Clinical Practice and the Declaration of Helsinki, and monitored by Karolinska Trial Alliance. The investigational procedure and the potential risks involved were explained to the patients orally and in writing at the pre-operative visit 2–28 days before surgery before obtaining their written informed consent by the investigator at the surgical ward the day before surgery.

### Study population

**Fig 1:**
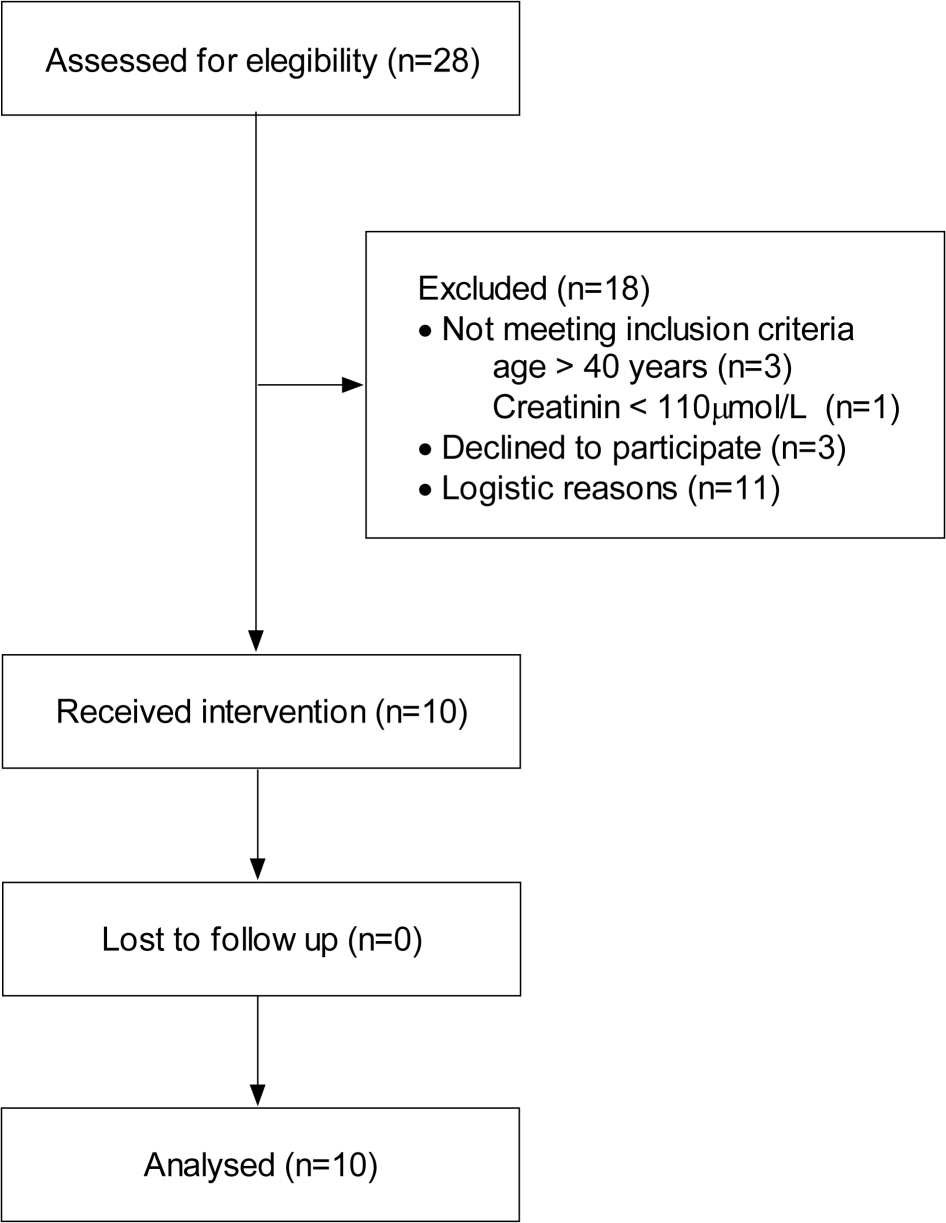
CONSORT diagram of patients. Logistic reasons for exclusion: Limits in research staff, more than one simultaneous pancreatic surgical procedure, and no available radioiodine isotope

Patients scheduled for pancreatic surgery at Karolinska University Hospital Huddinge were considered eligible. Ten of these were recruited during September to December 2010, and the study procedures including follow-up were completed within that time span. Because patients served as their own controls, we did not attempt to include consecutive subjects. Age over 40 years, plasma creatinine < 110 μmol/L, and negative pregnancy test for females, were required. Patient characteristics are presented in Table 1, and the CONSORT flowchart in Fig 1.

**Table 1:** Patient characteristics. Values are presented as median (range) or mean ± sd. ASA, American Society of Anesthesiologists.

Gender (Males: Females)	6: 4
Age (years)	64 (44–69)
Height (cm)	173 ± 10
Weight (kg)	73 ± 12
Body mass index (kg/m^2^)	24.4 ± 2.7
Weight loss (kg/last 3 months)	0 (0–5)
Plasma creatinine (μmol/L)	67 ± 19
ASA status (II:III)	4: 6

Three patients had pancreatic adenocarcinoma, three had intraductal papillary mucinous neoplasm of the pancreas, and the remaining four patients had gastrointestinal stromal tumour, neuroendocrine carcinoma, pancreatic cyst, or chronic pancreatitis with pain syndrome, respectively. Five patients were treated for hypertension, two had insulin dependent diabetes mellitus, two patients received pre-operative chemotherapy, one had steroids for sarcoidosis, and one patient was treated by high dose opioids for a chronic pain syndrome.

### Procedures

#### TER and Plasma Volume determination

**Fig 2:**
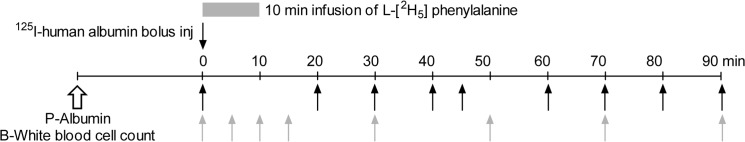
Flow chart of blood sampling, identical between day 0 and day 2. Black arrows: sampling of ^125^I-human serum albumin for determination of plasma volume and transcapillary escape rate. Gray arrows: sampling of l-[^2^H_5_] phenylalanine-albumin and l-[^2^H_5_] phenylalanine (precursor pool) for determination of albumin fractional synthesis rate.

In the morning after an overnight fast, patients were transferred to a pre-operative unit where they had intravenous and arterial lines inserted. The study drug, iodinated (^125^I) human albumin (SERALB-125, CIS bio international, Gif-sur-Yvette Cedex, France), was given as an intravenous injection of 100 kBq by an investigator. Arterial blood was sampled at 0, 20, 30, 40, 45, 60, 70, 80 and 90 min for determination of TER and plasma volume (Fig 2), which is well after the time of complete mixing[19]. An identical procedure was carried out on the morning of the second post-operative day at a high dependency surgical ward, although at this time the dose was increased to 300 kBq to compensate for the increased baseline. The calculated absorbed radiation dose was less than 0.3 mSv, and according to World Health Organization administration of potassium iodine is not indicated to protect the thyroid gland at such low doses for individuals over 40 years [20].

Blood was sampled in tubes with sodium heparin, and centrifuged at room temperature at 2000 g. Each weighted plasma sample was analysed by scintillation counting (Wallac Compugamma CS 1282, energy window 20–82 keV) during 10 to 60 min per sample to achieve a total count of at least 10.000 disintegrations to keep the error caused by variability in counts below 1%. TER was assessed as the slope of the linear regression of the logarithms of scintillation disintegrations per minute versus time plot [19]. Plasma volume was determined from dose and the back extrapolation of the same regression line to the time of injection [19, 21]. Intravascular albumin mass is the product of plasma volume and P-Alb, and albumin mass flow rate is the product of TER and intravascular albumin mass.

#### Albumin Fractional Synthesis Rate (FSR)

Immediately after the injection of iodinated human albumin, a 10 min intravenous infusion was started comprising l-[ring-^2^H_5_] phenylalanine (99 atom percent; Cambridge Isotope Laboratories Inc, Tewsbury, MA) dissolved in sterile water, together with unlabelled phenylalanine (Ajinomoto Company Inc, Tokyo, Japan), to an enrichment of 10 molar percent excess (100*concentration of tracer/(tracer+tracee)) and a total concentration of 20 mg/ml. Blood was sampled at 0, 5, 10, 15, 30, 50, 70 and 90 min for determination of FSR (Fig 2). The total volume of sampled blood amounted to 100 ml. An infusion with 20 atom percent excess of l-[^2^H_5_] phenylalanine was used at the similar procedure in the morning of the second post-operative day. Plasma samples were stored at −80°C until analysis. The details of sample preparation have been described previously in great detail [14, 15]. Briefly, albumin was separated from other protein constituents in plasma by repeated acid precipitation, washing, and dissolving in absolute ethanol. Thereafter, samples were hydrolyzed with 6 M HCl at 110°C for 24 hours, and dehydrated by vacuum desiccation. The phenylalanine in the hydrolysate was then enzymatically converted to beta-phenylethylamine, and the enrichment of l-[^2^H_5_] phenylalanine was calculated from the ratio of ions at m/z 180 and 183 of the t-butyldimethylsilyl derivative of phenylethylamine measured on an Agilent 5973 gas chromatograph mass spectrometer (Agilent Technologies, Santa Clara, CA) in comparison to a standard curve. Free phenylalanine enrichment in plasma was analysed after acid precipitation, cation-exchange chromatography, and monitoring the ions at m/z 336 and 341 of the t-butyldimethylsilyl derivative.

Albumin FSR were calculated from the slope of the enrichment of l-[^2^H_5_] phenylalanine in plasma albumin versus time plot divided by the area under the curve for the plasma precursor pool as suggested by Ballmer et al. [22]. Blood samples for determination of P-Alb were taken in EDTA-vials, spun at 2000 g, and then plasma was transferred to polypropylene vials and frozen at -20°C until analysed by an immunochemical method, nephelometry, at Studiecenter Karolinska University Hospital Solna. Intravascular albumin mass is the product of plasma volume and P-Alb. Absolute synthesis rate (ASR) was calculated by multiplying FSR with intravascular albumin mass and dividing by body weight [14, 22].

#### Anaesthesia

The anaesthetic procedure was carried out according to the local routines of our department. Briefly, premedication consisted of oral oxycodone and paracetamol. All subjects received a thoracic epidural catheter before start of general anaesthesia. A continuous epidural infusion of 12–15 ml per hour comprising bupivacaine (1 mg/ml), fentanyl (2 μg/ml), and epinephrine (2 μg/ml) was immediately started after catheter insertion [23].

General anaesthesia was induced by propofol, fentanyl and atracurium and maintained with sevoflurane (Sevorane, Abbot Scandinavia AB, Solna, Sweden) by approximately 0.8 minimal alveolar concentration in oxygenated air. A central venous line was inserted according to unit routines. Continuous intravenous fluids were given as infusions of Ringer’s solution (2 ml/kg/h; Ringer-Acetat, Fresenius Kabi, Uppsala, Sweden), starch (60 mg/ml, 2 ml/kg/h; Volulyte, Fresenius Kabi, Uppsala, Sweden) and Glucose (2.5 mg/ml, 1 ml/kg/h) throughout the surgical procedure. In this pragmatic study, bleeding substitution or extra fluid was given at the discretion of the attending anaesthetist. To maintain mean arterial blood pressure above 70 mmHg a continuous infusion of norepinephrine (40 μg/ml) was given, and maintained post-operatively until circulatory stability without vasopressor support was achieved. After end of surgery all patients were extubated in the operating room and surveyed in the recovery ward until next day, when they were transferred to a high dependency surgical ward for the rest of the study time. Postoperative pain was treated with continuous utilisation of the epidural catheter. All patients received intravenous infusion of glucose (5%) approximately 2 L/d. Ringer’s acetate and starch were provided to keep diuresis and blood pressure according to the discretion of the attending surgeon. No enteral nutrition was provided, and oral intake of water was limited to a few hundred ml. Patients were mobilised to stand along their bed and walk a short distance with aid if possible.

### Statistics

The primary outcomes were FSR, ASR and TER. Secondary outcomes were SIRS criteria, CRP, P-Alb, plasma volume, body weight and relationships between these and albumin kinetic parameters. With 10 evaluable patients there is a power of 80% to detect a difference of effect size of 1.0 (relevant change/ standard deviation of that change) with a 2-sided t-test and a significance level of 5%. This corresponds to a change in ASR with a standard deviation of 30 mg/kg/day and a difference of 30 mg/kg/day, values that fall in between volunteers and ICU patients in previous investigations. Data are presented as mean ± standard deviation or median (range) as appropriate. Outcome parameters are presented as mean (95% confidence interval). Paired samples were analysed by paired t-test if normality could not be ruled out according to Sharpio-Wilk’s test for normality, or Wilcoxon’s matched pairs test if data were non-parametric. Significance was determined at p < 0.05. The software STATISTICA 10 (StatSoft Inc. Tulsa, OK, USA) was used for statistical calculations.

## Results

### General management and anaesthesia

The only reported adverse event in this study was a mild itching of the eyes for 15 min after injection of the radio labelled iodine on the second study day in one single subject. All subjects received a norepinephrine infusion during anaesthesia, and in six patients this infusion was continued post-operatively for 17 (11–36) hours. Thus, in all patients norepinephrine infusion was stopped several hours before the measurement procedure on day 2. At the second measurement, the patients were investigated in a state of SIRS as presented in Table 2, where data represents values immediately before the isotope administration. The highest plasma C-reactive protein values were reached day 2 in 6 patients, and on day 3 in 4 patients. The highest white blood cell counts were reached on day 1 in 3, day 2 in 4, and day 3 in 3 patients. Analgesia was achieved by continuous epidural infusions, and on day 2 the patients scored 3 (2–5) on a numerical rating scale where 10 is worst imaginable pain.

**Table 2:** Physiological data before and 2 days after major pancreatic surgery, n = 10. Values are presented as mean (95% CI) or median (range), and differences analysed by ^1^paired t-test or ^2^Wilcoxon’s matched pairs test, respectively.

	Day 0	Day 2	Difference	p
^a^ Temperature (°C)	36.6 (36.4, 36.9)	37.8 (37.5, 38.2)	1.2 (0.8, 1.6)	< 0.001 ^1^
^a^ Respiratory rate (per min)	13 (11–19)	16.5 (15–20)	3.5 (-1–5)	0.008 ^2^
^a^ Heart rate (beats per min)	67 (59, 76)	84 (74, 94)	17 (6, 27)	0.007 ^1^
^a^ White blood cell count (10^9^/L)	5.9 (5.0, 6.9)	12.5 (9.1, 15.8)	6.5 (2.8, 10.2)	0.003 ^1^
C-reactive protein (mg/L)	0.9 (0.5–16.2)	127 (25–234)	124 (24–227)	0.005 ^2^
Haemoglobin (g/L)	138 (126, 149)	103 (97, 109)	-34 (-46, -22)	< 0.001 ^1^
Mean arterial pressure (mmHg)	82 (74, 90)	87 (78, 96)	5 (-6, 16)	0.33 ^1^
Body weight (kg)	72.9 (64.2, 81.6)	75.7 (67.0, 84.3)	2.8 (1.4, 4.1)	0.001 ^1^

^a^ Systemic inflammatory response syndrome parameter.

### Albumin kinetic parameters

**Fig 3:**
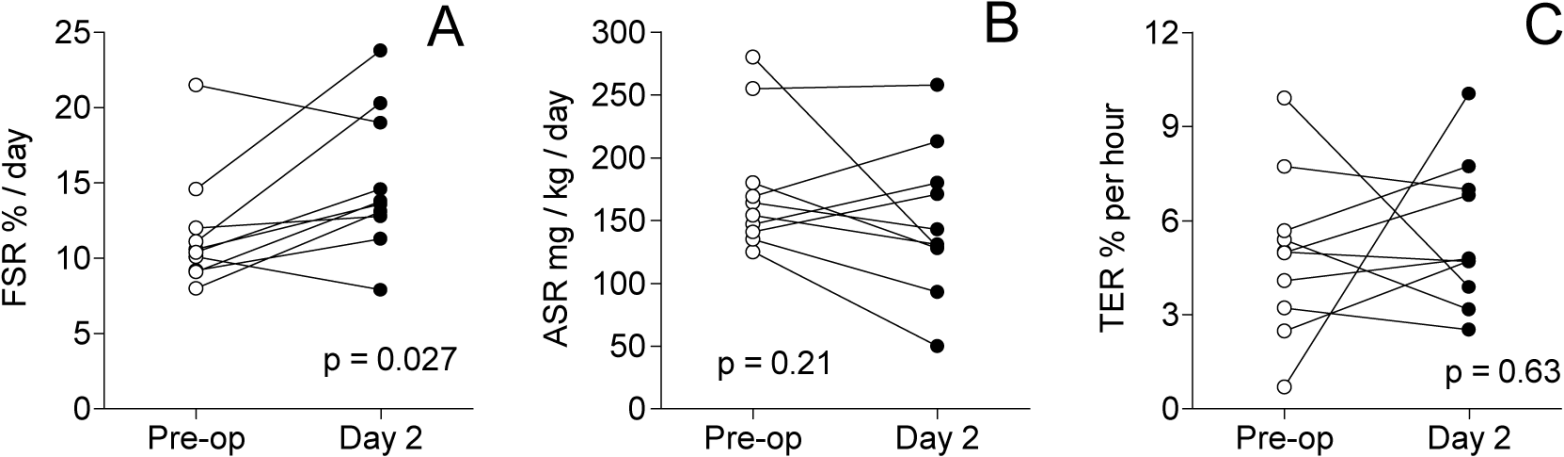
Albumin kinetic parameters in surgical patients assessed by flooding technique with d5-phenylalanin and dilution of iodinated human serum albumin, n = 10. A: Fractional synthesis rate (FSR), B: Absolute synthesis rate (ASR), Panel C: Transcapillary escape rate (TER). Measurements are performed immediately before (open circles) and two days after major pancreatic surgery (closed circles).

P-Alb decreased from baseline 33.5 (30.9, 36.2) to 22.1 (19.8, 24.3) g/L (p<0.001) two days after surgery. The calculated albumin kinetic parameters are presented in Table 3. The main finding was an increase in FSR from 11.7 (8.9, 14.5) to 15.0 (11.7, 18.4) % per day (p = 0.027, Fig 3), but the corresponding ASR was not altered significantly (p = 0.21, Fig 3). A documented increase in blood pressure in 5 of 10 patients, which correlated with the transition of patients to the operating room, was associated with an unphysiological elevation of plasma radioactivity and an erroneous decrease in TER. Therefore, only values until 60 min were used for calculation of TER and plasma volume. TER was not significantly different between the 2 study days (p = 0.63). Plasma volume was unchanged between study days whereas hematocrit decreased from 0.42 ± 0.04 to 0.32 ± 0.03, (p<0.001) which corresponded to a significant decrease in blood volume. Body weight was increased by 2.8 ± 1.9 kg on the second day after surgery (p < 0.001).

**Table 3:** Albumin kinetic parameters. Values presented as mean (95% confidence interval), before and 2 days after major pancreatic surgery, n = 10. Plasma volume and transcapillary escape rate were calculated from the slope 0–60 min after isotope injection. Blood volume was calculated from plasma volume and hematocrit assuming an F-ratio of 0.91.

	Day 0	Day 2	Difference	p
Plasma albumin concentration [g/L]	33.5 (30.9, 36.2)	22.1 (19.8, 24.3)	-11.5 (-13.3, -9.6)	< 0.001
Plasma volume [L]	3.31 (2.84, 3.78)	3.32 (2.91, 3.73)	0.01 (-0.33, 0.35)	0.97
Blood volume [L]	5.16 (4.44, 5.88)	4.45 (3.87, 5.02)	-0.72 (-1.15, -0.28)	0.005
Intravascular albumin mass [g]	110 (95, 138)	73 (61, 85)	-37 (-45, -29)	< 0.001
Fractional synthesis rate [%/d]	11.7 (8.9, 14.5)	15.0 (11.7, 18.4)	3.4 (0.5, 6.2)	0.027
Absolute synthesis rate [mg/kg/d]	175 (138, 212)	150 (107, 192)	-25 (-67, 17)	0.21
Transcapillary escape rate [%/h]	4.9 (3.1, 6.8)	5.5 (3.9, 7.2)	0.6 (-2.1, 3.4)	0.63
Albumin mass flow rate [g/h]	5.1 (3.6, 6.7)	3.9 (2.8, 5.1)	-1.2 (-3.4, 1.1)	0.26

**Fig 4:**
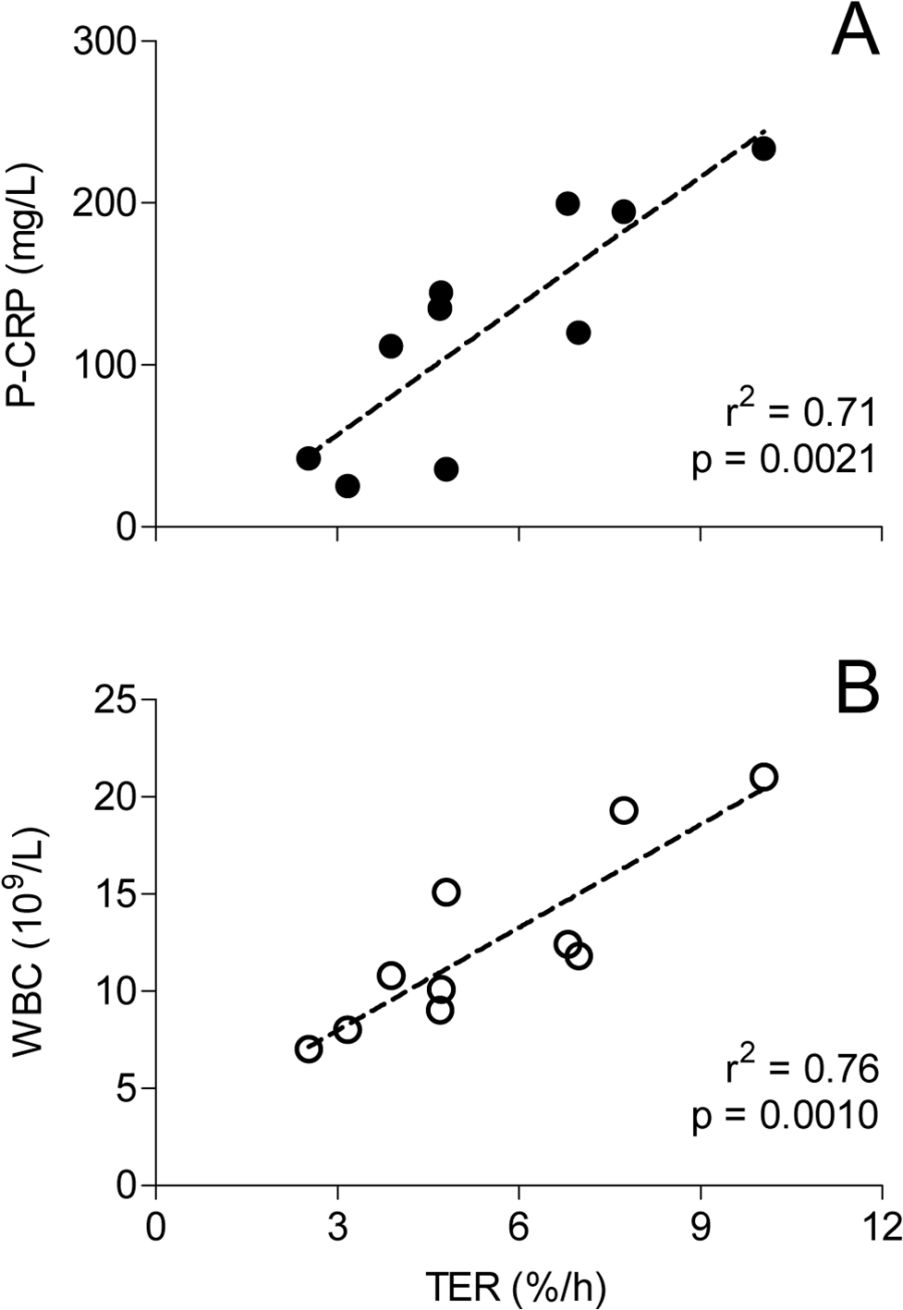
Correlation between albumin transcapillary escape rate (TER) and inflammatory parameters 2 days after major pancreatic surgery, n = 10. A: TER versus C-reactive protein, B: TER versus white blood cell count (WBC).

P-Alb did not correlate significantly to albumin kinetic parameters (p≥0.09), nor did TER and FSR correlate at any time point (p≥0.25). Post-operative day 2 TER was correlated to plasma C-reactive protein (r^2^ = 0.72, p = 0.0021) and white blood cell count (r^2^ = 0.76, p = 0.0010), respectively (Fig 4).

### Surgery and fluid balance

Seven patients had pancreaticoduodenectomy and one each pancreatectomy, duodenectomy, and a combination of fundoplication and splenectomy where the planned pancreatic resection of a cyst was postponed due to bleeding. Surgery time was 358 (208–598) min, and estimated bleeding 800 (500–3700) ml. Post-operative bleeding collected in drains was 584 ± 244 ml from end of surgery to the isotope measurement day 2. Five patients received erythrocyte infusions, together 12 units (225 ml each) during surgery, and 5 more units post-operatively. Likewise 8 units of plasma (250 ml each) were given to 3 patients (1+1+6 units) during and immediately after surgery and 2 units to a fourth patient day 1. Albumin infusions were given to two other patients between the two isotopic measurement days. In total, 6 patients received between 9 and 51 g of albumin between the two measurements, whereas losses in drains and by bleeding were 26 g (13–85). When compared to the decrease in intravascular albumin mass between the two study time points (Table 3) a cumulative albumin loss of −19 ± 12 g remains unexplained by this balance calculation, and is hypothetically the net leakage to the extravascular compartment.

The total amount of crystalloids given between the two isotopic measurements was 8.74 ± 0.76 L and the amount of colloids 2.65 ± 0.68 L, which together with blood products, bleeding and urine amounted to a calculated mean cumulative positive fluid balance of 5.8 ± 1.2 L. This is 3 kg more than expected from the corresponding change in body weight, but is partly explained by the insensible losses in the operating room, and during approximately 36 hours postoperatively.

## Discussion

### Albumin synthesis

In this pragmatic study, albumin kinetics was investigated immediately before and two days after major abdominal surgery, when inflammation reaches its peak in terms of white blood cell count and C-reactive protein. P-Alb was found to fall by 34 ± 6% from values that even before surgery were below the normal range.

Albumin FSR increased, whereas ASR was unaltered, 175 (138, 212) versus 150 (107, 192) mg/kg/day, p = 0.21, because of the concomitant decrease in P-Alb. In healthy subjects ASR is reported to be in the order of 194 mg/kg/d, based on studies on both synthesis and degradation in steady state conditions [24]. Very few publications address albumin synthesis rates in connection with surgery, and only two of these report repeated measurements in the same subjects [25, 26]. A decrease in both FSR and ASR is seen after 50 minutes of laparoscopic surgery compared to pre-operative values [25]. In infants, investigated between 8 and 16 hrs after admission to a paediatric intensive care unit after craniofacial surgery, ASR is high, around 250 mg/kg/d, but unaltered related to two different glucose infusion rates [26]. Following colorectal cancer surgery, ASR values between 125 and 300 mg/kg/d are reported on the second postoperative day related to different protocols of preoperative feeding [17]. Following major rectal surgery, FSR is reported higher on day 8–10, compared to the same patients 4 months later and to healthy controls [16]. The same investigators also report that a hyperglycemic hyperinsulinemic clamp fails to increase albumin synthesis in postoperative surgical patients [27].

### TER

In contrast to the finding of a 100% increase in TER 3–8 hours after cardiac surgery [12], we were unable to demonstrate an increased leakage on the 2^nd^ day after major abdominal surgery compared to the preoperative level. The large variation in the first TER measurement could partly be explained by factors associated with increased TER such as hypertension and cancer [12], but also to the fact that only 5 measurement points were used for the determination of TER, which is a limit of the study. The time schedule of this study was chosen to hit the peak of SIRS and two inflammatory markers that often occurs on day two after major pancreatic surgery. Furthermore, we wanted the first tracer dose to be reasonably mixed throughout the body compartments to get a stable baseline on the second measurement [28]. Although these goals were met, on average no increase in capillary leakage was apparent in our subjects. Either there is no such difference, or the study was underpowered to detect it. However, albumin leakage is reported to precede the acute phase protein response to surgery by at least a few hours [12]. Perhaps, other earlier markers of the inflammatory cascade are more synchronized with an increase in TER. Our data show an increase in body weight and fluid balance, and a loss of intravascular albumin indicative of a capillary leakage between the two measurements. Therefore, we speculate that by the second day, the increased TER associated with the surgical insult is returning to normal, and can no longer be demonstrated. This might, to some part, relate to the decreased albumin concentration gradient between plasma and the extracellular space. Very little is known about the exact concentrations of extracellular albumin in surgical patients, but in volunteers levels of 5–25 g/L are reported in leg lymph by lymph vessel cannulation [29]. By micro dialysis tissue levels of 13.2 g/L are reported in skeletal muscle of volunteers [30]. This leaves a narrow span of concentration driving force from the postoperative levels of plasma albumin in our patients.

There was a wide range (24–234 mg/L) in plasma C-reactive protein 2 days after pancreatduodenectomy. This suggests that the inflammatory response might be very variable in this group of patients, contributing to our failure to demonstrate an average capillary leakage of the same magnitude as reported immediately after thoracic surgery [12]. The apparent correlation between TER and biomarkers of inflammation, yet overall lack of significant change in TER, may represent a variation in the time course of capillary leakage between patients. This apparent inconsistency may also reflect that other factors, such as the presence of overhydration, may be equally important for the degree and duration of increased TER, and we would advise caution not to over interpret data in this pilot study. This, however, needs to be further explored.

### P-albumin, albumin balance and fluid balance

The assessment of plasma volume was very robust. Anthropometric blood volume [31] recalculated to plasma volume by hematocrit and an assumed correction factor between peripheral and central hematocrit of 0.91 showed a very close agreement with our measurements. The profound decrease in P-Alb seems to be partly explained by extravasation of albumin, because 19 ± 12 g of albumin is missing in the mass balance calculation based on the decrease in intravascular albumin mass (37 ± 11 g) compared to the sum of given and lost albumin. Similar findings of loss of plasma proteins have been reported in gynecological surgery [32]. The administration of starch, which at the time of the study was routinely used in our unit, and possible overhydration as suggested by increased body weight, might have affected both albumin and water distribution.

In this study we were unable to show any difference in albumin kinetic parameters of distribution (TER) and synthesis rate (ASR) 2 days after major pancreatic surgery compared to immediate preoperative baseline values. One contributing reason was that the standard deviations of both ASR and TER were larger than anticipated, causing the study to become underpowered, which is a limitation of the study. Plasma volume was also preserved, whereas P-Alb decreased. Measurement of albumin kinetic parameters during and immediately after end of the surgical procedure might better capture changes from baseline.

## Supporting Information

S1 TREND Checklist TREND Checklist. TREND statement checklist, N/A is not applicable.
(PDF)

S1 Protocol Study Protocol in English. (PDF)

S2 Protocol Study Protocol in Swedish. (PDF)

S1 Table Individual data. Data for all individual subjects in Tables 1–3, extra anthropometry, mass balance and fluid balance.
(PDF)
