# Supplementary material for: Albumin Kinetics in Patients Undergoing Major Abdominal Surgery
Source: PLoS One. 2015 Aug 27;10(8):e0136371. doi: 10.1371/journal.pone.0136371 (PMC4552033; doi:10.1371/journal.pone.0136371)
Supplement: S1 Protocol — (PDF) [file pone.0136371.s002.pdf]

ALBUMIN KINETICS IN GENERAL INFLAMMATION

An exploratory study in patients undergoing major abdominal surgery

FINAL

Product:	*SERALB-125*
Substance:	^{125}*I-labeled human serum albumin*
EudraCT-number:	*2010-018529-21*
Sponsor:	*Åke Norberg*<br>*Karolinska University Hospital, Huddinge*<br>*Dept of Anesthesia- and Intensive Care*
Study type:	*Open explorative fase IV study*
Version:	*1.0*
Date:	*2010-03-15*

1 TABLE OF CONTENTS

1 TABLE OF CONTENTS 2
2 SUMMARY 4
3 ABBREVIATIONS 5
4 ADMINISTRATIVE INFORMATION 6
4.1 SPONSOR AND PRIMARY INVESTIGATOR 6
4.2 CO-INVESTIGATORS AND OTHER PARTICIPATING RESEARCHERS 6
4.3 MONITORING 6
5 BACKGROUND 7
5.1 FLUID TREATMENT AND CHOICE OF THERAPHY 7
5.2 INFLAMMATION 7
5.3 ALBUMIN 7
6 STUDY AIM 8
7 RESEARCH QUESTION 8
7.1 PRIMARY QUESTION 8
7.2 SECONDARY QUESTION 8
8 ENDPOINTS 8
8.1 PRIMARY ENDPOINTS 8
8.2 SECONDARY ENDPOINTS 8
9 DESIGN 9
9.1 DESIGN OVERVIEW 9
10 ASSESSMENTS AND PROCEDURES 9
10.1 DAY -14 (DAY -28 TO -2). PATIENT INFORMATION 9
10.2 DAY -1 (DAY -3 TILL -1) – CONSENT AND SCREENING 9
10.3 DAY 0 – DAY OF SURGERY, RADIOIODINE FIRST DOSE 9
10.4 DAY 2 (DAY 1 TO 4) – RADIODINE SECOND DOSE 10
10.5 DAY 5 (DAY 4 TO 7) – FOLLOW UP 10
11 METHODS AND HANDLING OF SAMPLES 10
11.1 METHOD 10
11.2 HANDLING OF SAMPLES 10
11.3 ANALYSIS 10
11.4 FLOW SCHEDULE 11
12 SUBJECTS 11
12.1 INCLUSION CRITERIA 11
12.2 EXCLUSION CRITERIA 11
13 CRITERIA FOR DISCONTINUATION 12
13.1 PATIENT CRITERIA 12
13.2 INVESTIGATORS CRITERIUA AND EVALUABILITY 12
13.3 SUBJECT LOGG 12

FINAL

14 TREATMENT ... 12
14.1 INVESTIGATIONAL MEDICAL PRODUCT ... 12
14.2 PACKING, LABELING AND HANDLING OF THE INVESTIGATIONAL MEDICAL PRODUCT 12
14.3 DRUG COUNTING ... 12
15 ASSESSMENT OF SAFETY AND EFFICACY ... 13
15.1 ASSESSMENT OF PATIENT SAFETY ... 13
15.2 ASSESSMENT OF ANALYSIS RESULTS ... 13
16 HANNDLING OF ADVERSE EVENTS ... 13
16.1 DEFINITIONS ... 13
16.2 ASSESSMENT OF ADVERSE EVENTS ... 14
17 REPORTING OF ADVERSE EVENTS ... 14
17.1 REPORTING ADVERSE EVENTS (AE) ... 14
17.2 REPORTING SERIOUS ADVERSE EVENTS (SAE) ... 14
17.3 REPORTING SUSPECTED UNPREDICTED SERIOUS ADVERSE EVENTS (SUSAR) ... 14
17.4 FOLLOW UP OF ADVERSE EVENTS ... 15
18 STATISTICS AND DATA MANAGEMENT ... 15
18.1 DATA MANAGEMENT ... 15
18.2 STATISTICAL ANALYSIS ... 15
18.3 DETERMINATION OF THE NUMBER OF SUBJECTS ... 16
19 ASSESSMENT TO SOURCE DATA ... 16
20 QUATLITY CHECK ... 16
20.1 MONITORING ... 16
21 ETHICS ... 17
21.1 ETHICAL REVIEW BOARD ... 17
21.2 ETHICAL CONDUCT OF THE STUDY ... 17
22 DATA MANAGEMENT AND ARCHIVE ... 17
22.1 CASE REPORT FORMS ... 17
22.2 ARCHIVE ... 17
23 FINANSIAL SUPPORT AND INSURENS ... 17
24 PUBLICATION OF RESULTS ... 17
25 REFERENCES ... 18
26 SIGNATURE ... 19
27 ATTACHMENTS ... 20
27.1 FLOW CHART ... 20

FINAL

2 SUMMARY

PROTOCOL IDENTITY AND AIM	
EudraCT-numBer:	2010-018529-21
Protocol titel:	Albumin kinetics in general inflammation
Study aim:	Explore how general inflammation affects syntesis rate and capillary leakage of albumin

INVESTIGATIONAL MEDICAL PRODUCT	
Product:	SERALB-125
Pharmaceutical form:	Solution for injection
Mode of administration:	Intravenous injection

METHODOLOGY	
Study design:	Open explorative study
Dose:	0,1+0,3 MBq
Primary research question:	Is albumin synthesis rate and capillary leakage affected by general inflammation?
Effect parameters:	Synthesis rate and capillary leakage
Safety parameters:	Vital parameters

INVESTIGATED POPULATION	
Study subjects:	Patients undergoing scheduled major abdominal surgery
Number:	Maximum 16

TIME PLAN	
First patient included:	Q2 2010
Last patient included:	Q4 2010
Last patient finalized:	Q1 2011

FINAL

3 ABBREVIATIONS

Abbreviation	Unit	Explanation
^{5}d-Phe		Deuterium labeled phenylalanine
AF	per min	Respiratory rate
APE		Atom percent excess
ASR	mg/kg/day	Absolute Synthesis Rate
BMI	kg/m^2	Body Mass Index
Bq		Bequerelle, radiation dose
CRF		Case report form
CRP		C-reactive protein
FSR	%/day	Fractional Synthesis Rate
J_{alb}	mg/kg/hour	Mass flow of albumin from plasma
LPK		White blood cell count
MAP	mmHg	Mean Arterial Pressure
PV	liter	Plasma volume
SIRS		Systemic Inflammatory Response Syndrome
TER	%/hour	Transcapillary Escape Rate

4 ADMINISTRATIVE INFORMATION

4.1 SPONSOR AND PRIMARY INVESTIGATOR

Åke Norberg, M.D., Ph.D.
Karolinska Institutet, CLINTEC
Department of Anesthesia- and Intensive Care
Karolinska University Hospital, Huddinge B31
141 86 Stockholm, Sweden
Tel: 08-585 851 59
Mobil: 073-966 11 52
Fax: 08-779 54 24
E-post: ake.norberg@karolinska.se

4.2 CO-INVESTIGATORS AND OTHER PARTICIPATING RESEARCHERS

Christian Kahlbom, M.D.
Anestesi- och intensivvårdskliniken
Karolinska University Hospital, Huddinge B31
141 86 Stockholm, Sweden

Jan Wernerman, M.D., Ph.D., Professor
Anestesi- och intensivvårdskliniken
Karolinska University Hospital, Huddinge B31
141 86 Stockholm, Sweden

Ralf Segersvärd, M.D., Ph.D.
Gastrocentrum kirurgi, Sektionen för övre abdominell kirurgi
Karolinska University Hospital, Huddinge B31
141 86 Stockholm, Sweden

Olav Rooyackers, M.Sc., Ph.D., Associate professor
Anestesi- och intensivvårdskliniken
Karolinska Universitetssjukhuset, Huddinge
141 86 Stockholm, Sweden

4.3 MONITORING

Karolinska Trial Alliance
Contact: Birgitta Strandberg
Hälsingegatan 43
171 76 Stockholm, Sweden

FINAL

5 BACKGROUND

5.1 FLUID TREATMENT AND CHOICE OF THERAPHY

Fluid therapy is important with regard to many different diseases. In anesthesia and intensive care an aggressive fluid therapy is a cornerstone in the treatment of sepsis, but is also very important during major surgery, with or without bleeding, and in connection with trauma. The choice of fluid in these situations have been discussed for decades, but consensus have not been reached. Firstly, there are different views on the amount of fluid to be given, and then whether colloids (infusions of albumin, dextran, starch, gelatin or plasma) are preferable to crystalloids (isotonic electrolyte solutions) or not. There are also strong controversies regarding the various colloids, and not least, about the benefits or risks of albumin. The literature is inconsistent in this regard, where albumin is highlighted as dangerous [Cochrane 1998], harmless [SAFE 2004, Cochrane 2004] or particularly valuable for certain ICU patients [Dubois 2006]. Some reasons for these different opinions are based on the unclear indication for albumin use, and the incomplete knowledge concerning the physiology and kinetics of albumin. This study is the first of a research program that focuses on clarifying some of these important points about the albumin physiology and kinetics. Ultimately, the aim is to design a larger study that provides answers to albumin role in anesthesia and intensive care, and thus the opportunity for an optimized fluid therapy for large groups of patients. There are no previous studies that measured leakage and rate of synthesis of albumin at the same time, much less repeated measures.

5.2 INFLAMMATION

General inflammation is strongly associated with sepsis, which is a condition with high mortality despite modern intensive care. In Sweden to conduct clinical trials in this patient group, however, suffers from problems concerning the written consent, as patients are often unable to provide one. A milder form of influence is systemic inflammatory response syndrome (SIRS), which is characterized by changes in body temperature, heart rate, respiratory rate and the amount of white blood cells in the blood (LPK). One difference between SIRS and sepsis is if there is an infection or not. Major surgery triggers a general inflammation, and it is well known that many of the phenomena observed in septic patients also occur after surgery or other trauma.

In general inflammation, albumin levels in blood plasma is often low, regardless of whether the condition is due to infection, trauma, surgery, or in critically ill patients in intensive care. Other proteins increase, however, so-called acute phase proteins. One explanation for the low plasma levels of albumin is that albumin molecules are escaping from the bloodstream through the capillary walls, capillary leak (TER), which can be measured with radioiodine labeled human serum albumin as the fraction of albumin in the blood that leaves the blood stream per unit time. In thoracic surgery has been shown that capillary leakage of albumin is increased by 100% within 7 hours after surgery [Fleck 1985]. In general acute inflammation associated with infectious disease such as pneumonia, elevated values of TER has been reported [Ballmer 94]. TER may also be affected by diabetes, hypertension, malignancy, liver cirrhosis and rapid intravenous infusion of crystalloids. As a model of sepsis or SIRS, volunteers have been given endotoxine intravenously. This triggers an influenza-like condition and some biochemical changes, but not enough that you should get a measurable change in albumin TER [van Eijk 2005].

5.3 ALBUMIN

Albumin is a medium size molecule, 69 kDa, and the predominant protein in plasma (40 g/l of total 70 g/liter). The half-life of albumin is 14-20 days, and it is synthesized in the liver. The synthesis occurs as a prealbumin whose tertiary structure is configured for export from the liver. The process from the start of synthesis to export takes approximately 30 minutes. Albumin has multiple physiological functions, represents 70% of the oncotic pressure of plasma and acts as a transport protein and endogenous scavenger. Lack of albumin can be acute or chronic. Acute shortage is associated with general edema, but in chronic deficiency (congenital analbuminemia) surprisingly moderate symptoms occurs [Koot 2004].

FINAL

Albumin synthesis can be determined and quantified with great certainty [Ballmer 1990]. We have previously shown that ICU patients with multiple organ failure have greatly increased albumin synthesis [Essen 1998]. Furthermore, patients who undergo cholecystectomy during acute cholecystitis have higher albumin synthesis than those operated on in quiet phase [Barle 2006]. There is an older study in only 4 patients, which found decreased synthesis during inflammation, but with a different method of measurement [Moshage 1987]: This is partly supported by a number of animal studies that mainly used indirect methods [Vincent 2009]. However, there are reasons to believe that albumin synthesis is increased in many patients with generalized inflammatory reaction. The mechanisms and time course of this increase is unknown.

6 STUDY AIM

The study aim is to identify changes in albumin kinetics in patients who develop a generalized inflammation after major abdominal surgery.

7 RESEARCH QUESTION

7.1 PRIMARY QUESTION

Are albumin synthesis rate and capillary leakage affected by generalized inflammation?

7.2 SECONDARY QUESTION

Do changes in albumin kinetic variables (P alb, FSR, ASR, TER and Jalb) correlate to the degree of SIRS or the increase in CRP?

Do albumin kinetic parameters correlate with each other before or after surgery?

Does pre-operative body weight loss correlate with pre-operative albumin kinetic parameters?

8 ENDPOINTS

8.1 PRIMARY ENDPOINTS

Fractional synthesis rate (FSR) and absolute synthesis rate (ASR) for albumin. Transcapillary escape rate of albumin (TER) and mass flow from the blood (Jalb). P-albumin, plasma volyme, body weight.

8.2 SECONDARY ENDPOINTS

SIRS-criteria (body temperature, respiratory rate, heart rate, B-LPK) including CRP, before and after surgery.
Albumin kinetic parameters (P-albumin, FSR, ASR, TER, and Jalb).
Body weight and plasma volume, before and after surgery.
Pre-operative loss of body weight.

FINAL

9 DESIGN

9.1 DESIGN OVERVIEW

The study is a prospective, open, explorative pilot study of surgical patients scheduled for major abdominal surgery, pancreatic resection. A low dose of radioactive iodine (SERALB-125), will be injected intravenously on two occasions to study the albumin kinetics. At the same occasion, a flooding dose of deuterium labeled phenylalanine will be given. The actual study time is estimated to be about four hours spread over two days. The kinetics will be monitored for 90 minutes after the injection has been given. The first dose will be injected on the day of surgery, the second about two days after surgery. All other treatment will be given according to routine clinical practice. Recruitment will continue until 10 evaluable patients have completed all measurements. The staff who perform the laboratory analyzes will be "blinded”.

10 ASSESSMENTS AND PROCEDURES

10.1 DAY -14 (DAY -28 TO -2). PATIENT INFORMATION

The patients undergo the routine preoperative assessment by the anesthetist and have a meeting with the surgeon. Routine blood tests before surgery is taken and submitted to hospital laboratory. These include the P-creatinine. No study-specific samples are taken at this time. Patients are informed orally and in writing about the study and the study-specific procedures of the investigator or co-investigator. The patient receives written study information to take home to read. Consent may be obtained at this visit for those patients who are willing to give it. However, probably the majority of patients give their consent at admittance to the hospital the day before surgery.

10.2 DAY -1 (DAY -3 TILL -1) – CONSENT AND SCREENING

The investigator visits the ward where the patient is admitted. The investigator will answer any newly emerging issues and then obtain the written consent. Women of childbearing capacity will have a U-pregnancy test. The investigator verifies that the blood samples taken at the -14 day visit are answered, and that the patient meets all inclusion criteria and no exclusion criteria exist. Chemotherapy or antibiotic treatment should be documented in the CRF. All other care given in the ward before surgery is standardized and according to local procedures. The patient's height and weight are documented in the CRF, as well as weight loss of 6 months. Now, It will be noted in the patient medical record that the patient participates in a clinical trial in accordance with LVFS 2003: 6, Chapter 3, 4§.

10.3 DAY 0 – DAY OF SURGERY, RADIOIODINE FIRST DOSE

In the morning of the day of surgery, the patient arrives to the pre-anesthesia ward about two hours before anesthesia startup (this is the "overtime" in addition to clinical practice) and meets an investigator and a research nurse who is well informed about the study. The usual measures before this kind of major surgery is performed in accordance with local routines, including an artery line and an intravenous peripheral catheter. Blood pressure, pulse and respiratory rate followed. Temperature, pulse and respiratory rate are documented in the CRF. Blood samples for determination of P-albumin and P-CRP are collected for analysis at Studiecenter at Karolinska University Laboratory, Solna. B-LPK is analyzed at the routine hospital laboratory. Then, the patient receives an injection of radioiodine labeled albumin (SERALB-125, 0.1 MBq). A flooding dose of isotope-labeled phenylalanine is supplied over 10 minutes. Blood samples (total 46 ml) are taken at 0, 5, 10, 15, 20, 30, 40, 45, 60, 70, 80 and 90 minutes after injection of radioiodine and the start of the flooding dose of isotope-labeled phenylalanine, for determining the enrichment of the isotope phenylalanine in the blood, for the measurement of capillary leakage of albumin, and for determination of plasma volume. Then, anesthesia, surgery and postoperative care are performed according to standard procedures at the Karolinska University Hospital Huddinge.

FINAL

10.4 DAY 2 (DAY 1 TO 4) – RADIODINE SECOND DOSE

The investigator is responsible for documentation of postoperative complications in the CRF. Any lack of evaluability is assessed and documented in the CRF(see paragraph 11.3). New P- albumin and P-CRP are received as described above, and respiratory rate, pulse and body temperature noted in the CRF. The same study procedure as "day 0 - day of operation" is implemented as a second series of measurements in the same way over 90 minutes with radioiodine labeled albumin and a flooding dose of phenylalanine, although at higher doses to compensate for the increased background from the first measurement (blood samples, a total of 41ml).

10.5 DAY 5 (DAY 4 TO 7) – FOLLOW UP

At least three days after the second series of measurements the investigator notes any complications in the CRF. A copy of monitoring hospital records are taken and patient identity concealed. These are attached to the CRF.

FINAL

11 METHODS AND HANDLING OF SAMPLES

11.1 METHOD

Measurement of radioactivity will be performed by scintillation counting at the Department of Nuclear Medicine, Karolinska Huddinge. Quantitative measurement of enrichment of isotope-labeled phenylalanine will be performed using gas chromatography-mass spectrometry at the Karolinska Stable Isotope Core where Olav Rooyackers is laboratory manager. Quantification of total phenylanalin (precursor) will also occur with mass spectrometry compared to an internal standard.

11.2 HANDLING OF SAMPLES

The plasma samples for the measurement of stable isotopes will get de-identified labels, and will be kept in the existing bio bank at the Department of Anesthesiology and Intensive Care, Karolinska University Hospital Huddinge at -80 °C pending completion of the data collection, and then analyzed in the same run to obtain the best possible accuracy in accordance with local standard operative procedures. Sample Collection Manager is Åke Norberg. All testing materials will be destroyed when the material is published. Passkey will be stored in a locked room at the Department of Anesthesiology, internal address B43. Responsible for this is Åke Norberg.

11.3 ANALYSIS

The step from fractional synthesis rate (FSR %/d) to absolute synteshanstighet (ASR mg/kg/d) is taken by multiplying FSR by the intravascular albumin mass (IAM, g), which in turn is a product of the P-albumin and plasma volume (PV) and finally dividing the result by the body weight. Similarly, the total mass flow rate of albumin from the blood (Jalb) is calculated from the transcapillary albumin leakage (TER).

11.4 FLOW CHART

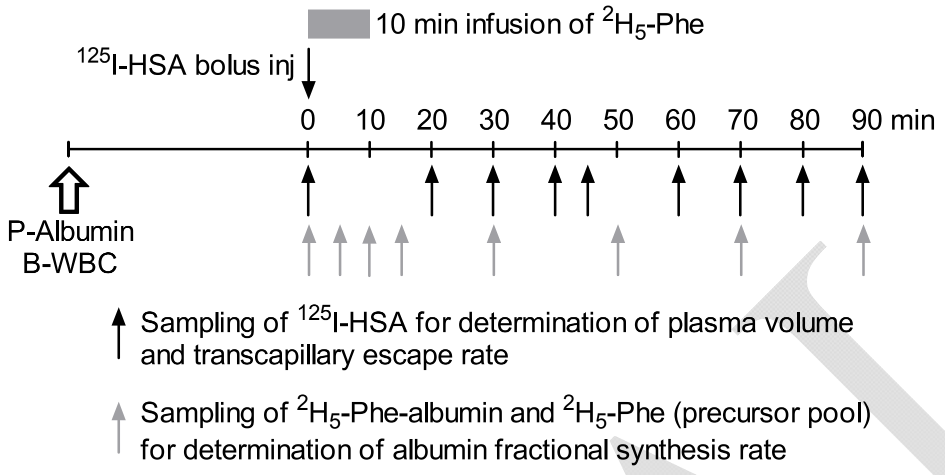

12 SUBJECTS

12.1 INCLUSION CRITERIA

- Patients scheduled for major abdominal surgery, pancreatic resection
- Males and females ≥ 40 years
- Written informed consent

12.2 EXCLUSION CRITERIA

- Pregnancy or breast feeding patients
- Patients planned for perioperative albumin/plasma infusion (primarily in chronic renal dysfunction but perhaps also in some kinds of coagulopathy)
- P-creatinine > 110
- Allergy to the investigational medical product
- Other circumstance that causes the investigator to judge the patients participation as unsuitable.

FINAL

13 CRITERIA FOR DISCONTINUATION

13.1 PATIENT CRITERIA

The patient may, at any time during the study, chose to end his/hers further participation in the study, without giving any cause. If possible, the investigator/nurse will record the reason for study end in the CRF.

13.2 INVESTIGATORS CRITERIA AND EVALUABILITY

The primary investigator can determine that the patient cannot stay in the study. This can be founded on lack of evaluability or the reasons stated below:

- Errors in technical equipment
- Staf shortage that makes safe and standardized methods impossible.
- Ongoing major bleeding
- Massiv transfusion of albumin or plasma

This shall be stated in the CRF with date and time.

13.3 SUBJECT LOG

The investigator will keep a record of all patients informed about the study on a log list. The log list is stored in the investigator brouschure available on Department of Anesthesiology. It will show included and not included subjects.

14 TREATMENT

14.1 INVESTIGATIONAL MEDICAL PRODUCT

SERALB-125. Solution for injection 1.7 ml. Contains 320 kBq ^{125}I-HSA (human serum albumin, corresponding to 100 µCi). The dose 0.1 and 0.3 MBq at the first and second occation, respectively, is given as an intravenous injection.

SERALB-125 is approved for this purpose in France. For more details see appendix 1 (SPC).

14.2 PACKING, LABELING AND HANDLING OF THE INVESTIGATIONAL MEDICAL PRODUCT

The investigational medical product is produced in France, and imported to Sweden by Lars Segerberg at **Electra-Box AB,** Box 2035, 135 02 Tyresö. Then the drug is transported to Karolinska University Hospital Huddinge, to hospital pharmacy, and finally to the Department of Nuclear Medicine. There radioactivity in plasma is assessed. The local routines for the documentation and delivery to medical wards of radioactive compounds will be applied. Disposal of surplus material and samples will be in accordance with local routines at Dept of Nuclear Medicine, Karolinska University Hospital, Huddinge.

14.3 DRUG COUNTING

The investigational medical product will be collected at the Department of Nuclear Medicine to each patient and the ampoule will be labeled in accordance with appendix 2.

Doses collected from Dept of Nuclear Medicine will be documented in special medicine log list that is available in the investigator binder. There, each patient’s dose is documented with date and time.

FINAL

15 ASSESSMENT OF SAFETY AND EFFICACY

The primary investigator has undergone radiation protection training (Appendix 3). A meeting with Lena Engelin (Radiation nurse Nuclear Medicine Karolinska Solna) and the employees to be included in the study team is planned before the start of the study, *i.e.* when the investigator receive MPA's approval. There everyone will be informed about the measures Radiation Protection Committee deems necessary for waste handling, labeling of test tubes, etc. Application to Radiation Protection Committee at Karolinska Huddinge and Ethical Review Board in Stockholm takes place in parallel with application to the MPA.

15.1 ASSESSMENT OF PATIENT SAFETY

Patients will be monitored in the surgical ward, pre-anesthesia ward and intermediate ward K44, resepctivley, for the time when SERALB-125 is injected by a staff that is familiar with emergency incidents. Monitoring of the patient's heart rate, ECG, respiratory rate, oxygen saturation (oximetry), blood pressure and temperature are taken before and during the first 90 min after injection.

15.2 ASSESSMENT OF ANALYSIS RESULTS

Laboratory samples taken before and after surgery (*i.e*. P-creatinine, P-CRP, B-LPK) will be analyzed at the ISO certified Karolinska University Laboratory, Huddinge. Blood gases are analyzed by alanyzers at the Department of Anesthesiology. P-albumin will be analyzed by immunochemical method through the Studiecenter at the Karolinska University Laboratory, Solna. Other samples are analyzed as described in paragraph 9.

16 MANAGEMENT OF ADVERSE EVENTS

16.1 DEFINITIONS

In such major surgical procedures that occur in this study, all patients will per- or postoperative encounter what in other contexts would be classified as AEs/SAEs. The surgery itself causes hospitalization (SAE). Anticipated effects of anesthesia or surgery will not be reported, even if they can be considered undesirable. These don not relate to the isotope and are not under the control of the investigator. However, such medical events that may affect the study results or the evaluability will be listed in the CRF.

16.1.1 Adverse events (AE)

An adverse event is defined as a medical event that occurs from the time the patient receives the isotope injection to 90 minutes thereafter.

16.1.2 Adverse reactions (AR)

An adverse reaction is a harmful and unintended medical response to a medical product. For an AE to become an AR, the suspected association between the product and the reaction must at least be possible.

16.1.3 Serious adverse events (SAE)

An unintended medical event that at any dose:

- Results in death
- Is life-threatening
- Causes hospitalization or prolongation of existing hospitalization
- Results in persistent or temporary disability
- Results in congenital damage or malformation

FINAL

- Is serious by other means

16.1.4 Suspected unpredicted serious adverse events (SUSAR)

A reaction or event that is unexpected, serious, suspected to be caused by the treatment and that is not previously described.

16.2 ASSESSMENT OF ADVERSE EVENTS

16.2.1 Assessment of severity

Each adverse event will be classified by the investigator as mild, moderate or severe.

Mild: The event does not affect the person's normal life.

Moderate: The event causes deterioration of function but does not affect health. The event gives discomfort and/or causes inconvenience.

Severe: The event causes deterioration of function or working capacity or endanger the health of the person.

16.2.2 Assessment of causality

Likely: Clinical event, including abnormal laboratory analyzes, which occur within a reasonable time after administration of the intervention / study product. Unlikely that the event can be attributed to underlying disease, other drugs or other treatment.

Possible: Clinical event, including laboratory analyzes, which occur within a reasonable time after administration of the intervention / study product. The event might be explained by underlying disease or other drugs.

Unlikely: Clinical event, including abnormal laboratory analyzes, which can temporarily be related to the administration of the intervention / study product. The event is unlikely related to the intervention / study product and can be explained by other drugs or underlying disease.

Not possible to classify: The event cannot be classified because of missing information or that the event is not verified.

17 REPORTING OF ADVERSE EVENTS

17.1 REPORTING ADVERSE EVENTS (AE)

All undesirable events that occur immediately adjacent to SERALB-125 dose should be considered as an AE and documented in accordance with ICH GCP principles.

17.2 REPORTING SERIOUS ADVERSE EVENTS (SAE)

Serious adverse events should be reported to the sponsor on a special SAE form within 24 hours of the investigator learned of the SAE. Follow-up information that describes the outcome and management of the SAE must be reported as soon as the information is available. The original document should be placed in the CRF.

17.3 REPORTING SUSPECTED UNPREDICTED SERIOUS ADVERSE EVENTS (SUSAR)

The sponsor is responsible for ensuring that all relevant information about suspected, unexpected, serious adverse reactions are recorded and reported to EudraVigilans and to relevant ethical review boards.
SUSARs that are fatal or life-threatening shall be reported to EudraVigilans as soon as possible and no

FINAL

later than 7 days after the incident has become known to the sponsor. Relevant follow-up data should then be forwarded within another 8 days. Other SUSARs shall be reported as soon as possible and no later than 15 days after they arrived at Sponsor's knowledge. For this type of reporting, the sponsor has contracted Karolinska Trial Alliance, which will be responsible for such reports.

17.4 FOLLOW UP OF ADVERSE EVENTS

The principal investigator (physician) will be present or immediately available during the whole experiment and will make assessment of the severity if adverse event occurs. Adverse events not related to the study, will be followed up within the routine framework of the clinic.

18 STATISTICS AND DATA MANAGEMENT

18.1 DATA MANAGEMENT

All laboratory values will be printed from the patient record (Take Care, TC) de-identified and attached to the CRF. All values of the vital parameters during and after the injection will be documented directly on a coded anesthesia paper record by the research nurse or the investigator. This journal is a part of the CRF. At the end of the surgical procedure, the anesthesia records will be copied and made anonymous and attached to the CRF. Other documents that are stored in the CRF for coding and de-identification are: pre-anesthetic assessment, medical record, medication list from TC, surgery report and monitoring records until 3 days after the second series of measurements with isotopes.

Data from the mass spectrometer measurements of phenylalanine will be exported as a data sheets via a USB stick.

All the data regarding end-points will be added in an Access database that is locked when data collection is complete.

De-identified and coded patient demographics preoperatively, per-operatively and post-operatively will be prepared and included in the Access database.

18.2 STATISTICAL ANALYSIS

There is evidence that the rate of synthesis of albumin, measured as fractional or absolute synthesis rate, increase in states of general inflammation that occurs after this kind of surgery. Furthermore, albumin capillary leakage increases as measured by the fraction of albumin in the blood and the total outflow of albumin from the blood. The endpoint is the change in these four parameters. To take the step from fractions to absolute values requires an assessment of plasma volume, P-albumin and body weight. All data will be presented as mean with standard deviation (SD) or as median and range of data if not normally distributed. The changes in the variables will be tested with two-sided paired t-test alternative Wilcoxon’s test with a significance level of 0.05.

Furthermore, the mutual correlations between the four kinetic albumin parameters as well as with P-albumin will be calculated and presented with correlation coefficients. That changes in capillary leakage and synthesis rate could correlate is reasonable, but not previously described.

Correlation between the above kinetic albumin variables and degree of SIRS (HR, LPK, temperature, respiratory rate) including CRP will be explored. It is reasonable to believe that the degree of inflammation affects the change of albumin variables. Influence of pre-operative weight loss on albumin preoperative kinetic variables will also be analyzed, because weight loss could be a factor that already pre-operatively affects albumin kinetic variables. Correlations will be reported as Pearson correlation coefficient r or Spearman's rank correlation rs depending on the data's nature.

Demographic preoperative data such as age, height, weight, BMI, gender, some routine lab, preoperative weight loss, duration of pancreatic disease, diagnoses, current medications, completing

FINAL

chemotherapy and ASA class will be presented as numbers, averages and standard deviation, or median range depending on the data's nature. Likewise per- and postoperative demographics such as surgery haemorrhage, supplied anesthetics including epidural anesthesia (doses, number), given fluid infusions including blood products (ml), surgical procedure, post-operative medical or surgical complications, postoperative drug delivery, including the epidural, and laboratory test according to routine will be reported.

18.3 DETERMINATION OF THE NUMBER OF SUBJECTS

In paragraph 1 of this protocol, we specify the total number of patients to 16. The higher number is a way to keep abreast of any shortfall, but the analysis are so costly that the intention is to cancel the recruitment when we reached the 10 evaluable patients.

With 10 evaluable patients there is a power of 80% to detect a difference of effect size 1 with a 2-sided t-test and a significance level of 5%. The absolute albumin synthesis rate has been measured at 131 ± 21 mg/kg/day in healthy subjects (control group) where intra-individual standard deviation for repeated measurement after 2.5 hours was 11 mg/kg/day [Barle 2002], which can be perceived as the uncertainty of the method in this specific and very controlled situation. With repeated measurements on ICU patients with 5-day intervals corresponding values were 233 ± 67 and 70 mg/kg/day [Barle 2001a]. There is reason to believe that in this case we will fall between these figures. If the variation of the change is 30 mg/kg/day a difference in the absolute synthesis rate of 30 mg/kg/d can be detected with 80% power. We believe this to be a clinically relevant change.

Some patients can be expected to have increased capillary leakage (TER) in the basal measurement (before surgery) due to hypertension, diabetes and malignancy, but this is less of a problem when the patients serve as their own controls.

Ten patients, give an 80% power to detect a correlation between, for example, capillary leak and synthesis rate if r is greater than 0.71 ($r^2 \geq 0.5$) with a two-sided test and a significance level of 5%.

We have not found any published measurements of neither synthesis rate or capillary leakage of albumin in this patient group. Any assumption regarding the distribution and change in albumin kinetic parameters thus becomes unsure. There is a risk that this pilot study proves to be a bit too weak in power, but it is very resource intensive measurements, and if so we can submit an amendment to expand the investigation. The large number of correlations mentioned above must be seen against the background that this is an exploratory pilot study of physiological changes in scheduled surgery. There is thus an intention that the study is mainly hypothesis generating.

19 ACCESS TO SOURCE DATA

The primary investigator is responsible that there is a confidentiality agreement between the monitor or any other independent reviewer (*i.e.* auditor) given permission to verify the data in the patient record and the patient record responsible party at Dept of Anesthesiology and Intensive Care. This requires the patient's written consent.

20 QUATLITY CHECK

20.1 MONITORING

The investigator with staff must set aside time for monitoring, audits, and regulatory inspections. The sponsor is responsible for monitoring by independent monitor with knowledge of GCP, to ensure that the study is performed in accordance with GCP, the study protocol and regulatory requirements. The sponsor has contracted Karolinska Trial Alliance for monitoring of this study.

FINAL

21 ETHICS

21.1 ETHICAL REVIEW BOARD

The investigator is responsible for the application for approval from the Ethics Review Board. Sponsor's responsibility is to send a copy of the approval to the Swedish MPA.

21.2 ETHICAL CONDUCT OF THE STUDY

The study will be conducted according to the study protocol, GCP, regulatory requirements and the Helsinki Declaration.

22 DATA MANAGEMENT AND ARCHIVE

22.1 CASE REPORT FORMS

Data collected in the study and registered in the CRF will be transferred to a database that the sponsor is responsible of.

Data collected in the study will be recorded in Case Report Forms (CRF) in which the patient is only identified with a code number and initials. The code key kept by the investigator in such a way that unauthorized persons cannot take part of it. Anonymous and coded copies of medical records such as anesthesia records, monitoring sheet for post-operative care, journal text (Take Care) and laboratory lists (Take Care) will also be included in the CRF.

22.2 ARCHIVE

Study documents will be filed at least 10 years after the study report is submitted to the Swedish MPA. The documents should be archived in a legible condition for eventual future audit or inspection by authorities.

23 FINANCIAL SUPPORT AND INSURANCE

The study is funded by research grants. The patients are insured through medical insurance and patient injury insurance.

24 PUBLICATION OF RESULTS

The intention is to publish the results in a scientific journal.

FINAL

25 REFERENCES

Ballmer PE, McNurlan MA, Milne E, et al. (1990). Measurement of albumin synthesis in humans: a new approach employing stable isotopes. Am J Physiol E797-803

Ballmer PE, Ochsenbein AF, Schütz-Hofmann S. (1994). Transcapillary escape rate of albumin positively correlates with plasma albumin concentration i acute but not in chronic inflammatory disease. Metabolism 43: 697-705

Barle H, Gamrin L, Essén P, et al. (2001a). Growth hormone does not affect albumin synthesis in the critically ill. Intensive Care Med 27: 836-43

Barle H, Råhlén L, Essén P, et al. (2001b). Stimulation of human albumin synthesis and gene expression by growth hormone threatment. Clin Nutr 20: 59-67

Barle H, Januszkiewicz A, Hållström L, et al. (2002). Albumin synthesis in humans increases immediately following the administration of endotoxin. Clin Sci 103: 525-31

Barle H, Hammarqvist F, Westman B, et al. (2006). Synthesis rates of total liver protein and albumin are both increased in patients with an acute inflammatory response Clin Sci 110, 93–9

Cochrane Injuries Study Group. (1998). Human albumin administration in critically ill patients: systematic review of randomised controlled trials. BMJ 317: 235-40

Cochrane Database Syst Rev (2004). Human albumin solution for resuscitation and volume expansion in critically ill patients. Okt 18 (4): CD001208.

Dubois M-J, Crellana-Jimenez C, Melot C, et al. (2006). Albumin administration improves organ function in critically ill hypoalbuminemic patients: a prospective, randomized, controller pilot study. Crit Care Med 34: 2536-40

Essen P, McNurlan MA, Gamrin L, et al. (1998). Tissue protein synthesis rates in critically ill patients. Crit Care Med 26: 92-100

Fleck A, Hawker F, Wallace PI, et al. (1985) Increased vascular permeability: a major cause of hypoalbuminaemia in disease and injury. Lancet 1: 781-4

Koot BGP, Houwen R, Pot D-J, et.al. (2004). Congenital analbuminaemia: biochemical and clinical implications. A case report and literature review. Eur J Pediatr 163: 664-70

Moshage HJ, Janssen JAM, Franssen JH, et al. (1987) Study of the molecular mechanism of decreased liver synthesis of albumin in inflammation. J Clin Invest 79: 1635-41

SAFE study investigators (2004). A comparison of albumin and saline for fluid resuscitation in the intensive care unit. New Eng J Med 350: 2247-56

van Eijk LTGJ, Pickkers P, Smits P, et al. (2005), Microvascular permeability during experimental human endotoxemia: an open intervention study. Crit Care 2005, 9: R157-64

Vincent JL. (2009) Relevance of albumin in modern critical care medicine. Best Pract Res Clin Anaesthesiol. 23:183-91

FINAL

26 SIGNATURE

This page should be signed by the sponsor and the primary investigator.

Primary Investigator
Åke Norberg
Dept. of Anesthesiology and Intensive Care
Karolinska University Hospital Huddinge

Signature | Date

Sponsor
Åke Norberg
Dept. of Anesthesiology and Intensive Care
Karolinska University Hospital Huddinge

Signatur | Date

Annan function
Name, titel

Signature | Date

FINAL

27 ATTACHMENTS

27.1 Flow chart

	DAY - 14 (DAY -28 TO -2)	DAY -1 (DAY -3 TO -1)	DAG 0	DAY 2 (DAY 1 TO DAY 4)	DAY 5 (DAY 4 TO DAY 7)
Investigator visit	X	X	X	X	X
Blood sampling ROUTINE	X		X	X	X
Oral study information	X	X			
Written patient information	X				
Written consent	(X)	X			
Inclusion/exclusion criteria		X			
Demographic data		X	X	X	X
Diseaseses		X		X	X
Ongoing medication		X		X	X
Blood sampling: P-Albumin, P-CRP			X	X	
Blood sampling: B-LPK			X		
Urine-HCG (women)		**X**			
Radiolabeled iodine injection, dose #1			X		
Radiolabeled iodine injection, dose #2				X	
Isotoplabeled injection of d5-phenylalanin			X	X	
Vital parameters (BP, AF, pulse, temp)		X	X	X	X
Adverse events			X	X	

FINAL
