# Supplementary material for: Albumin Kinetics in Patients Undergoing Major Abdominal Surgery
Source: PLoS One. 2015 Aug 27;10(8):e0136371. doi: 10.1371/journal.pone.0136371 (PMC4552033; doi:10.1371/journal.pone.0136371)
Supplement: S2 Protocol — (PDF) [file pone.0136371.s003.pdf]

ALBUMINKINETIK VID GENERELL INFLAMMATION

En explorativ studie på patienter vid stor bukkirurgi

FINAL

Produkt:	*SERALB-125*
Substans:	*^{125}I-märkt humant serum albumin*
EudraCT-nummer:	*2010-018529-21*
Sponsor:	*Åke Norberg*<br>*Karolinska Universitetssjukhuset, Huddinge*<br>*Anestesi- och intensivvårdskliniken*
Typ av studie:	*Öppen explorativ fas IV studie*
Version:	*1.0*
Datum:	*2010-03-15*

1 INNEHÅLLSFÖRTECKNING

1 INNEHÅLLSFÖRTECKNING 2

2 SAMMANFATTNING 4

3 FÖRKORTNINGAR 5

4 ADMINISTRATIV INFORMATION 6

4.1 SPONSOR OCH ANSVARIG PRÖVARE 6
4.2 MEDPRÖVARE OCH ÖVRIGA MEDVERKANDE FORSKARE 6
4.3 MONITORERING 6

5 BAKGRUNDSINFORMATION 7

5.1 VÄTSKEBEHANDLING OCH TERAPIVALSPROBLEM 7
5.2 INFLAMMATION 7
5.3 ALBUMIN 7

6 STUDIENS SYFTE 8

7 FRÅGESTÄLLNING 8

7.1 PRIMÄR FRÅGESTÄLLNING 8
7.2 SEKUNDÄR FRÅGESTÄLLNING 8

8 ENDPOINTS 8

8.1 PRIMÄRA ENDPOINTS 8
8.2 SEKUNDÄRA ENDPOINTS 8

9 DESIGN 9

9.1 ÖVERSIKTLIG DESIGN 9

10 BEDÖMNINGAR OCH PROCEDURER 9

10.1 DAG -14 (DAG -28 TILL -2). PATIENTINFORMATION 9
10.2 DAG -1 (DAG -3 TILL -1) – SAMTYCKE OCH SCREENING 9
10.3 DAG 0 – OPERATIONSDAG, RADIOJOD FÖRSTA DOS 9
10.4 DAG 2 (DAG 1 TILL 4) – RADIOJOD ANDRA DOSEN 10
10.5 DAG 5 (DAG 4 TILL 7) – UPPFÖLJNING 10

11 METOD OCH PROVHANTERING 10

11.1 METOD 10
11.2 PROVHANTERING 10
11.3 ANALYS 10
11.4 FLÖDESSCHEMA 11

12 FÖRSÖKSPERSONER 11

12.1 INKLUSIONSKRITERIER 11
12.2 EXKLUSIONSKRITERIER 11

13 KRITERIER FÖR AVBRYTANDE 12

13.1 PATIENTKRITERIER 12
13.2 PRÖVARENS KRITERIER OCH EVALUERBARHET 12
13.3 FÖRSÖKSPERSONSLOGG 12

FINAL

14 BEHANDLING 12
14.1 BESKRIVNING AV FÖRSÖKSLÄKEMEDLET 12
14.2 PACKNING, MÄRKNING OCH HANTERING AV FÖRSÖKSLÄKEMEDLET 12
14.3 LÄKEMEDELSRÄKNING 12
15 BEDÖMNING AV SÄKERHET OCH EFFEKTIVITET 13
15.1 BEDÖMNING AV PATIENTSÄKERHET 13
15.2 BEDÖMNING AV ANALYSSVAR 13
16 HANTERING AV OÖNSKADE MEDICINSKA HÄNDELSER 13
16.1 DEFINITIONER 13
16.2 BEDÖMNING AV OÖNSKADE MEDICINSKA HÄNDELSER 14
17 RAPPORTERING AV OÖNSKADE MEDICINSKA HÄNDELSER 14
17.1 RAPPORTERING AV OÖNSKADE MEDICINSKA HÄNDELSER (AE) 14
17.2 ALLVARLIGA OÖNSKADE MEDICINSKA HÄNDELSER (SAE) 14
17.3 RAPPORTERING AV MISSTÄNKTA, OFÖRUTSEDDA, ALLVARLIGA OÖNSKADE REAKTIONER (SUSAR) 14
17.4 UPPFÖLJNING AV OÖNSKADE MEDICINSKA HÄNDELSER 15
18 STATISTIK OCH DATAHANTERING 15
18.1 DATAHANTERING 15
18.2 STATISTISK ANALYS 15
18.3 BESTÄMNING AV ANTAL FÖRSÖKSPERSONER 16
19 TILLGÅNG TILL KÄLLDATA 16
20 KVALITETSKONTROLL 16
20.1 MONITORERING 16
21 ETIK 17
21.1 ETIKPRÖVNINGSNÄMNDEN 17
21.2 ETISKT GENOMFÖRANDE AV STUDIEN 17
22 DATAHANTERING OCH ARKIVERING 17
22.1 CASE REPORT FORMS 17
22.2 ARKIVERING 17
23 FINANSIERING OCH FÖRSÄKRING 17
24 PUBLICERING AV RESULTAT 17
25 REFERENSER 18
26 SIGNATUR 19
27 BILAGOR 20
27.1 FLÖDESSCHEMA 20

2 SAMMANFATTNING

**PROTOKOLLIDENTITET OCH MÅLSÄTTNING**	
EudraCT-nummer:	2010-018529-21
Protokolltitel:	Albuminkinetik vid generell inflammation
Studiens målsättning:	Undersöka hur generell inflammation påverkar synteshastighet och kapillärläckage av albumin

**PRÖVNINGSLÄKEMEDEL**	
Produkt:	SERALB-125
Farmaceutisk form:	Lösning
Administreringssätt:	Intravenös injektion

**METODOLOGI**	
Studiedesign:	Öppen explorativ studie
Dos:	0,1+0,3 MBq
Primär frågeställning:	Påverkas albuminets synteshastighet och kapillärläckage av generell inflammation
Effektivitetsparametrar:	Synteshastighet och kapillärläckage
Säkerhetsparametrar:	Vitala parametrar

**FÖRSÖKSPERSONSPOPULATION**	
Försökspersoner:	Elektiv stor bukkirurgi
Antal:	Maximalt 16

**TIDSPLAN**	
Första patienten inkluderas:	Q2 2010
Sista patienten inkluderas:	Q4 2010
Sista patienten avslutas:	Q1 2011

FINAL

3 FÖRKORTNINGAR

Förkortning	Enhet	Förklaring
^{5}d-Phe		Deuteriummärkt fenylalanin
AF	per minut	Andningsfrekvens
APE		Atom percent excess
ASR	mg/kg/dag	Absolute Synthesis Rate (absolut synteshastighet)
BMI	kg/m^2	Body Mass Index
Bq		Bequerelle, stråldos
CRF		Studieprotokoll för enskild patient (Case report form)
CRP		C-reaktivt protein
FSR	%/dag	Fractional Synthesis Rate (fraktionell synteshastighet)
J_{alb}	mg/kg/timme	Massutflöde av albumin från plasma
LPK		Vita blodkroppar
MAP	mmHg	Mean Arterial Pressure (medelartärblodtryck)
PV	liter	Plasmavolym
SIRS		Systemic Inflammatory Response Syndrome
TER	%/timme	Transcapillary Escape Rate (kapillärläckage)

4 ADMINISTRATIV INFORMATION

4.1 SPONSOR OCH ANSVARIG PRÖVARE

Åke Norberg, M.D., Ph.D.
Karolinska Institutet, CLINTEC
Vid Anestesi- och intensivvårdskliniken
Karolinska Universitetssjukhuset, Huddinge B31
141 86 Stockholm
Tel: 08-585 851 59
Mobil: 073-966 11 52
Fax: 08-779 54 24
E-post: ake.norberg@karolinska.se

4.2 MEDPRÖVARE OCH ÖVRIGA MEDVERKANDE FORSKARE

Christian Kahlbom, M.D.
Anestesi- och intensivvårdskliniken
Karolinska Universitetssjukhuset, Huddinge
141 86 Stockholm

Jan Wernerman, M.D., Ph.D., Professor
Anestesi- och intensivvårdskliniken
Karolinska Universitetssjukhuset, Huddinge
141 86 Stockholm

Ralf Segersvärd, M.D., Ph.D.
Gastrocentrum kirurgi, Sektionen för övre abdominell kirurgi
Karolinska Universitetssjukhuset, Huddinge
141 86 Stockholm

Olav Rooyackers, M.Sc., Ph.D., Associate professor
Anestesi- och intensivvårdskliniken
Karolinska Universitetssjukhuset, Huddinge
141 86 Stockholm

4.3 MONITORERING

Karolinska Trial Alliance
Kontaktperson: Birgitta Strandberg
Hälsingegatan 43
171 76 Stockholm

FINAL

5 BAKGRUNDSINFORMATION

5.1 VÄTSKEBEHANDLING OCH TERAPIVALSPROBLEM

Vätskebehandling är en viktig åtgärd vid många olika sjukdomstillstånd. Inom anestesi- och intensivvård utgör en aggressiv vätsketerapi en av hörnstenarna i behandlingen av sepsis, men är också mycket viktigt vid större operationer, med eller utan blödning, och i samband med trauma. Valet av vätsketerapi vid denna typ av behandling har diskuterats under årtionden utan att man kommit fram till en entydig konsensus. Dels finns det skilda uppfattningar om mängderna vätska som ska ges, dels om användning av kristalloider (isotona elektrolytlösningar) jämfört med kolloider (infusioner av albumin, dextran, stärkelse, gelatin eller plasma). Det finns också starka kontroverser kring de olika kolloiderna och inte minst kring nyttan eller riskerna med albumin. Litteraturen är härvidlag motsägelsefull där albumin framhållits som farligt [Cochrane 1998], ofarligt [SAFE 2004, Cochrane 2004] eller särskilt värdefullt för vissa intensivvårdspatienter [Dubois 2006]. Troligen grundar sig en del av åsiktsskillnaderna i att indikationen för albuminanvändningen har varit oklar, samt att det finns en ofullständig kunskap beträffande fysiologi och kinetik av bland annat albumin. Denna studie är den första i ett forskningsprogram som fokuserar på att klarlägga några av dessa viktiga punkter kring albumins fysiologi och kinetik. Målet på sikt är att designa en större studie som ger svar på albuminets roll inom anestesi och intensivvård och därmed möjlighet till en optimerad vätskebehandling för stora patientgrupper. Det finns inga tidigare studier där man mätt läckage och synteshastighet för albumin samtidigt, än mindre vid upprepad mätning.

5.2 INFLAMMATION

Vid generell inflammation tänker man i första hand på sepsis, som är ett tillstånd med mycket hög mortalitet trots modern intensivvård. Att i Sverige genomföra kliniska prövningar i denna patientgrupp är dock behäftat med problem kring skriftligt samtycke, eftersom patienterna ofta är ur stånd att lämna ett sådant. En något mildare form av påverkan är systemiskt inflammatoriskt svarssyndrom (SIRS) som karaktäriseras av förändringar i kroppstemperatur, puls, andningsfrekvens och mängden vita blodkroppar i blod (LPK). Skillnaden mellan SIRS och sepsis ligger i om det samtidigt finns en infektion eller inte. Stor kirurgi utlöser en generell inflammation, och det är välkänt att många av de fenomen som ses hos septiska patienter också förekommer efter kirurgi eller annat trauma.

Vid generell inflammation är albuminnivåerna i blodplasma ofta låga, oavsett om tillståndet beror på infektion, trauma, kirurgi, eller hos kritiskt sjuka patienter inom intensivvård. Andra äggviteämnen ökar däremot, så kallade akuta fasproteiner. En del av förklaringen till de låga plasmanivåerna av albumin är att albuminmolekylerna läcker ut från blodbanan genom kapillärväggarna, kapillärläckage (TER), vilket kan mätas med radiojodmärkt albumin som den fraktion av albumin i blodet som lämnar blodbanan per tidsenhet. Vid thoraxkirurgi har man visat att kapillärläckaget för albumin ökar med 100 % inom 7 timmar efter kirurgi [Fleck 1985]. Vid generell akut inflammation i samband med t ex pneumoni har man sett förhöjda värden på TER [Ballmer 94]. TER kan också påverkas av diabetes, hypertoni, malignitet, levercirrhos och hastig intravenös infusion av kristalloider. Som en modell för sepsis eller SIRS används ibland frivilliga forskningspersoner som får endotoxin intravenöst. Det utlöser ett influensaliknande tillstånd och en del biokemiska förändringar, men räcker inte för att man ska få en mätbar förändring av TER för albumin [van Eijk 2005].

5.3 ALBUMIN

Albumin är en medelstor molekyl, 69 kDa, och utgör det dominerande äggviteämnet i plasma (40 g/liter av totalt 70 g/liter). Normalt omsätts albumin med en halveringstid på 14–20 dagar, och det syntetiseras i levern. Syntesen sker som ett prealbumin vars tertiärstruktur konfigureras för export från levern. Processen från start av syntes till export tar cirka 30 minuter. Albumin har flera fysiologiska funktioner, utgör 70 % av det onkotiska trycket i plasma, fungerar som transportprotein och som endogen scavanger. Brist på albumin kan vara akut eller kronisk. Vid akut brist uppstår generellt ödem, men vid kronisk brist (kongenital analbuminemi) förekommer förvånansvärt måttliga symtom [Koot 2004].

Albuminsyntesen kan bestämmas och kvantifieras med stor säkerhet [Ballmer 1990]. Vi har själva tidigare visat att intensivvårdspatienter med multipel organsvikt har en kraftigt förhöjd albuminsyntes

[Essen 1998]. Vidare har patienter som opereras akut för gallstenssjukdomen under pågående gallblåseinflammation högre albuminsyntes än de som opereras i lugnt skede [Barle 2006]. Det finns en äldre studie på endast 4 patienter, där man fann minskad syntes vid inflammation, men med en annan mätmetod [Moshage 1987], något som till viss del stöds av ett antal djurstudier där man huvudsakligen använt indirekta mätmetoder [Vincent 2009]. Det finns dock skäl att tro att albuminsyntesen är ökad hos många patienter med generell inflammatorisk reaktion. Mekanismerna och tidsförloppet för denna ökning är okänt.

6 STUDIENS SYFTE

Studien syftar till att kartlägga förändringar i albuminkinetiken hos patienter som utvecklar en generell inflammation efter stor bukkirurgi.

7 FRÅGESTÄLLNING

7.1 PRIMÄR FRÅGESTÄLLNING

Påverkas albuminets synteshastighet och kapillärläckage av generell inflammation?

7.2 SEKUNDÄR FRÅGESTÄLLNING

Korrelerar graden av SIRS respektive ökningen av CRP med värden eller förändringar i albumins kinetiska variabler (P-alb, FSR, ASR, TER och Jalb)?

Korrelerar albumins kinetiska variabler med varandra före och efter kirurgi?

Korrelerar pre-operativ viktförlust med pre-operativa kinetiska variabler för albumin?

8 ENDPOINTS

8.1 PRIMÄRA ENDPOINTS

Fraktionell synteshastighet (FSR) och absolut synteshastighet (ASR) för albumin. Procentuellt kapillärläckage av albumin (TER) respektive massutflöde (Jalb). P-albumin, plasmavolym, vikt.

8.2 SEKUNDÄRA ENDPOINTS

SIRS-kriterier (kroppstemperatur, andningsfrekvens, hjärtfrekvens B- LPK) inklusive CRP, före och efter kirurgi.
Albumins kinetiska variabler (P-albumin, FSR, ASR, TER och Jalb) samt kroppsvikt och plasmavolym före och efter kirurgi.
Pre-operativ viktförlust.

FINAL

9 DESIGN

9.1 ÖVERSIKTLIG DESIGN

Studien är en prospektiv, öppen, explorativ pilotstudie av kirurgiska patienter som planerats för stor bukkirurgi, pancreasresektion. En lågdos av radioaktiv jod (SERALB-125), injiceras intravenöst vid två tillfällen för att studera albuminkinetiken. Vid samma tillfällen ges också en flödesdos av deuteriummärkt fenylalanin. Den faktiska studietiden beräknas till ca fyra timmar fördelade på två dagar. Kinetiken följs under 90 minuter efter det att injektion givits. En dos injiceras på operationsdagen den andra ca två dagar efter operation. I övrigt sker all annan behandling i enlighet med klinisk praxis. Rekryteringen fortsätter till 10 evaluerbara patienter genomgått samtliga mätningar. Personalen som utför de laborativa analyserna kommer att vara ”blindade”.

10 BEDÖMNINGAR OCH PROCEDURER

10.1 DAG -14 (DAG -28 TILL -2). PATIENTINFORMATION

Patienterna genomgår sedvanlig preoperativ bedömning av anestesiolog och inskrivningsssamtal med kirurg. Sedvanliga blodprover inför kirurgi tas och lämnas till rutinlab. Där ingår bland annat P-kreatinin. Inga studiespecifika prover är aktuellt vid denna tidpunkt. Patienterna informeras muntligen och skriftligen om studien och de studiespecifika procedurerna av prövaren eller medprövare. Patienten får en skriftlig patientinformation att ta med sig hem att läsa. Samtycke kan inhämtas vid detta besök för de patienter som önskar så. Sannolikt kommer dock majoriteten av patienterna lämna sitt samtycke vid inkomst till sjukhus dagen innan operation.

10.2 DAG -1 (DAG -3 TILL -1) – SAMTYCKE OCH SCREENING

Prövaren besöker vårdavdelning där patienten är inlagd. Prövaren besvarar eventuella nyuppkomna frågor och inhämtar därefter det skriftliga samtycket. Fertila kvinnor lämnar U-graviditets test. Prövaren kontrollerar att de blodprover som togs vid besök -14 är besvarade, och att patienten uppfyller samtliga inklusionskriterier samt att inga exklusionskriterier föreligger. Om patienten varit föremål för cytostatikabehandling eller antibiotika behandling skall detta dokumenteras i CRF. All annan vård som ges på avdelningen innan operation är standardiserad och följer de lokala rutinerna. Patientens längd och vikt dokumenteras i CRF, liksom viktnedgång 6 månader. Här sker också sedvanlig journalföring i befintligt journalsystem att patient medverkar i klinisk studie i enlighet med LVFS 2003:6, 3 kap 4§.

10.3 DAG 0 – OPERATIONSDAG, RADIOJOD FÖRSTA DOS

På operationsdagens morgon anländer patienten ca två timmar innan anestesistart (detta är ”mertid” utöver klinisk praxis) och tas emot av prövare och sköterska som är välinformerad om studien. Sedvanliga åtgärder inför denna typ av operation utförs i enlighet med lokala rutiner, bland annat sätts en artärnål och en intravenös infart. Blodtryck, puls och andningsfrekvens följs. Temperatur, puls och andningsfrekvens dokumenteras i CRF. Blodprov för bestämning av P-albumin och P-CRP tas för analys via Studiecenter vid Karolinska Universitetslaboratoriet, Solna. B-LPK tas för bestämning på rutinlab. Patienten erhåller därefter en injektion av radiojodmärkt albumin (SERALB-125, 0,1 MBq). En flödesdos av isotopmärkt fenylalanin tillförs under 10 minuter. Blodprover (totalt 46 ml) tas vid 0, 5, 10, 15, 20, 30, 40, 45, 60, 70, 80 samt 90 minuter efter injektion av radiojod och start av flödesdos av isotopmärkt fenylalanin, för bestämning av berikning av isotopmärkt fenylalanin i blodet, för mätning av kapillärläckage för albumin och för bestämning av plasmavolym. Därefter genomförs anestesi, kirurgi och postoperativ vård enligt gällande rutiner på Karolinska Universitetssjukhuset Huddinge.

FINAL

10.4 DAG 2 (DAG 1 TILL 4) – RADIOJOD ANDRA DOSEN

Prövaren ansvarar för att dokumentera om några postoperativa komplikationer inträffat. Dessa dokumenteras i CRF. Eventuellt bristande evaluerbarhet värderas och dokumenteras (se punkt 11.3). Nytt P-albumin och P-CRP tas som ovan, och andningsfrekvens, puls och temperatur noteras i CRF. Samma studieprocedur som *”dag 0 - operationsdag”* genomförs som en andra mätserie på samma sätt över 90 minuter med radiojodmärkt albumin och en flödesdos av fenylalanin, men med högre doser för att kompensera för den ökade bakgrunden från den första mätningen (blodprover totalt 41 ml).

10.5 DAG 5 (DAG 4 TILL 7) – UPPFÖLJNING

Prövaren dokumenterar att det gått 3 dygn sedan andra mätserien i CRF och noterar om någon komplikation inträffat sedan föregående besök. Kopia av övervakningsjournalen tas och avidentiferas. Denna bifogas till CRF.

11 METOD OCH PROVHANTERING

11.1 METOD

Mätning av radioaktivitet kommer att ske med scintillationsräknare vid avdelningen för Nuklearmedicin, Karolinska Huddinge. Kvantitativ mätning av berikning av isotopmärkt fenylalanin görs med hjälp av gaskromatografi-masspektrometri på Karolinska Stable Isotope Core där Olav Rooyackers är laboratoriechef. Kvantifiering av total-phenylanalin (precursorn) kommer också att ske med masspektromer mot intern standard.

11.2 PROVHANTERING

Provmaterialet för mätning av stabila isotoper kommer att märkas på ett avidentifierat sätt och förvaras i befintlig biobank på Anestesi- och Intensivvårdskliniken Karolinska Universitetssjukhuset Huddinge vid -80°C i väntan på att materialinsamlingen är avslutad och sedan analyseras i samma analysomgång för erhållande av bästa möjliga precision i enlighet med lokala rutiner. Provsamlingsansvarig är Åke Norberg. Allt provtagningsmaterial kommer att destrueras när materialet är publicerat. Kodnyckel kommer att förvaras i låst utrymme på anestesiavdelningen internadress B43. Ansvarig för detta är Åke Norberg.

11.3 ANALYS

Steget från fraktionell synteshastighet (FSR, %/d) till absolut synteshanstighet (ASR, mg/kg/d) tas genom att FSR multipliceras med intravaskulär albuminmassa (IAM, g) vilket i sin tur är en produkt av P-albumin och plasmavolymen (PV) och slutligen divideras resultatet med kroppsvikten. På samma sätt kan man från det transkapillära albuminläckaget (TER) beräkna totalt massutflöde av albumin från blodbanan (Jalb).

FINAL

11.4 FLÖDESSCHEMA

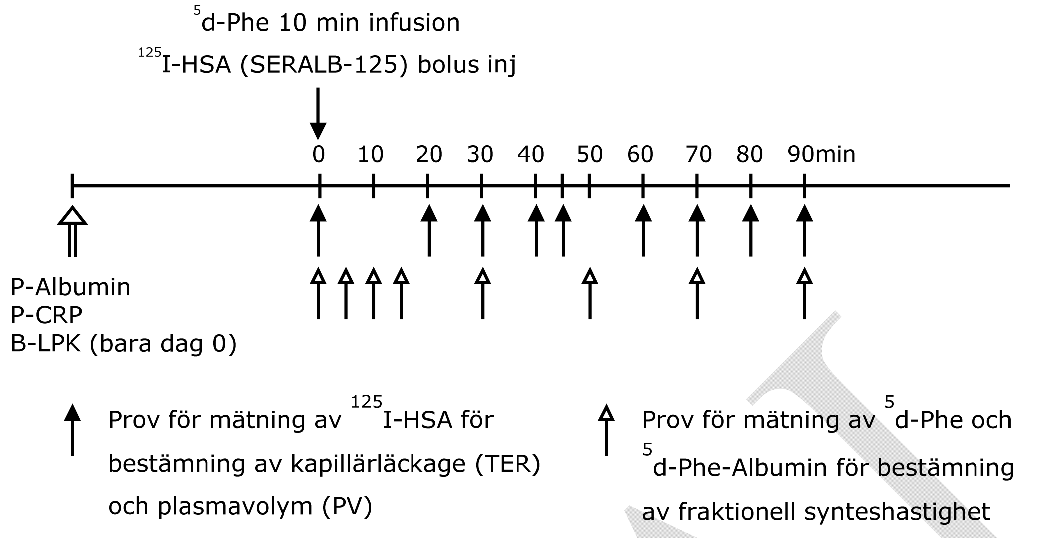

12 FÖRSÖKSPERSONER

12.1 INKLUSIONSKRITERIER

- Patienter planerade för stor elektiv bukkirurgi, pankreasresektion
- Män och kvinnor ≥ 40 år
- Signerat skriftligt samtycke

12.2 EXKLUSIONSKRITERIER

- Gravida eller ammande patienter
- Planerad för perioperativ albumin/plasma tillförsel (detta är i första hand aktuellt vid njurinsufficiens, men också vid vissa typer av koagulationsrubbningar)
- P-kreatinin > 110
- Allergi mot prövningsläkemedlet
- Annan omständighet som gör att ansvarig prövare bedömer patientens deltagande såsom olämpligt

FINAL

13 KRITERIER FÖR AVBRYTANDE

13.1 PATIENTKRITERIER

Patienten kan när som helst under studiens gång avbryta sitt deltagande i studien utan att uppge orsak.

Om möjligt skall prövaren/sköterskan dokumentera anledningen till studieslut i CRF.

13.2 PRÖVARENS KRITERIER OCH EVALUERBARHET

Huvudprövaren kan själv göra bedömningen att patienten inte kan fortsätta i studien. Detta kan orsakas av bristande evaluerbarhet eller andra skäl redogjorda för nedan:

- Tekniskt fel på utrustning
- Personella skäl som omöjliggör säker och standardiserad metodanalys
- Pågående stor blödning
- Massiv tillförsel av albumin eller plasma

Dessa skall dokumenteras i CRF med datum och klockslag.

13.3 FÖRSÖKSPERSONSLOGG

Prövaren kommer att dokumentera alla patienter som varit på screeningbesök i en logglista. Logglistan förvaras i prövarpärm som finns på anestesiexpeditionen. Där framgår vilka som inkluderats samt vilka som inte inkluderats.

14 BEHANDLING

14.1 BESKRIVNING AV FÖRSÖKSLÄKEMEDLET

SERALB-125. Injektionsvätska 1,7 ml. Innehåller 320kBq ^{125}I-HSA (humant serumalbumin, motsvarar 100 µCi). Dosering: 0,1 + 0,3 MBq vid första respektive andra mätningen ges som intravenös injektion.

SERALB-125 är godkänt för denna användning i Frankrike. För utförligare beskrivning se bifogad bilaga 1. (SpC).

14.2 PACKNING, MÄRKNING OCH HANTERING AV FÖRSÖKSLÄKEMEDLET

Prövningsläkemedlet produceras i Frankrike och tas in i Sverige av partihandlare Lars Segerberg på **Electra-Box AB,** Box 2035, 135 02 Tyresö. Därefter kommer läkemedlet att transporteras till Karolinska Universitetssjukhuset Huddinge, till sjukhusapoteket för slutlig transport till avdelningen för Nuklearmedicin. Där mäts och analyseras plasmaaktivitet av SERALB-125. Dessa har också tydliga regler och dokumentationsrutiner för hur detta skall dokumenteras vid uthämtning till klinik. Destruktion av överblivet material kommer att ske i enlighet med befintliga rutiner på Nuklearmedicin, Karolinska Huddinge.

14.3 LÄKEMEDELSRÄKNING

Försöksläkemedlet kommer att hämtas på avdelningen för nuklearmedicin inför varje patient som har inkluderas och ampullen kommer att märkas med etikett, se bifogad bilaga 2.

Alla doser som hämtas från nuklearmedicin dokumenteras på särskild läkemedelslogg lista som finns tillgänglig i prövarpärm. Där framgår tydligt vilken patient som erhållit vilken dos samt datum och tidpunkt.

FINAL

15 BEDÖMNING AV SÄKERHET OCH EFFEKTIVITET

Huvudprövaren har genomgått en strålskyddsutbildning (bilaga 3). Ett möte med Lena Engelin (strålskyddssjuksköterska Nuklearmedicin Karolinska Solna) och de medarbetare som skall ingå i studieteamet, planeras inför start av studien, dvs när prövaren fått Läkemedelsverkets godkännande. Där skall alla få information om de åtgärder som strålskyddskommittén anser nödvändiga för sophantering, uppmärkning av provrör etc. Ansökan till Strålskyddskommittén vid Karolinska Huddinge och Etikprövningsnämnden i Stockholm sker parallellt med ansökan till Läkemedelsverket.

15.1 BEDÖMNING AV PATIENTSÄKERHET

Patienter kommer att övervakas på operationsavdelningen respektive intermediärvårdsavdelning K44 under den tid som SERALB-125 ges av personal som är väl förtrogen med akuta händelser. Monitorering av patientens hjärtfrekvens, EKG, andningsfrekvens, saturation (oximetri), blodtryck och temperatur görs innan och under de första 90 min efter injektionen.

15.2 BEDÖMNING AV ANALYSSVAR

Laboratorieprover som tas före och efter operation, (t ex P-kreatinin, P-CRP, B-LPK) analyseras på det ISO-certifierade Karolinska Universitetslaboratoriet, Huddinge. Blodgaser analyseras patientnära på respektive avdelnings egna blodgasapparater. P-albumin analyseras med immunokemisk metod via Studiecentrets vid Karolinska Universitetslaboratoriet, Solna försorg. Övriga prover analyseras enligt beskrivningen punkt 9.

16 HANTERING AV OÖNSKADE MEDICINSKA HÄNDELSER

16.1 DEFINITIONER

Vid den typ av större kirurgi som förekommer i denna studie kommer alla patienter att per- eller post-operativt råka ut för vad som i andra sammanhang skulle klassas som AE/SAE. Kirurgin som sådan orsakar t ex sjukhusvistelse (SAE). Förväntade effekter av anestesi eller kirurgi kommer därför inte att rapporteras, även om de kan betraktas som oönskade. Dessa relaterar inte till isotopen och ligger inte under prövarens kontroll. Däremot kommer sådana medicinska händelser som kan tänkas påverka studieresultatet eller evaluerbarheten att noteras i CRF.

16.1.1 Oönskade medicinska händelser (AE)

En oönskad händelse definieras som en medicinsk händelse som inträffar från det att patienten erhåller isotopinjektionen till 90 minuter därefter.

16.1.2 Oönskade reaktioner (AR)

En oönskad reaktion är en skadlig och oavsiktlig medicinsk reaktion på en medicinsk produkt. För att en AE ska bli en AR ska den misstänkta associationen mellan produkt och reaktion vara åtminstone ***möjlig***.

16.1.3 Allvarliga oönskade medicinska händelser (SAE)

Varje oavsiktlig medicinsk händelse som vid någon dos:

- resulterar i död
- är livshotande
- föranleder sjukhusvistelse eller förlängd sjukhusvistelse

FINAL

- resulterar i kvarstående eller tillfälliga handikapp
- resulterar i en medfödd skada/missbildning
- Allvarlig av annan orsak

16.1.4 Misstänkta, oförutsedda, allvarliga oönskade reaktioner (SUSAR)

En reaktion/händelse som är oväntad, allvarlig, misstänks vara orsakad av behandlingen och som inte finns beskriven tidigare.

16.2 BEDÖMNING AV OÖNSKADE MEDICINSKA HÄNDELSER

16.2.1 Bedömning av svårighetsgrad

Varje oönskad medicinsk händelse ska klassificeras av prövaren som mild, moderat eller svår.

Mild: Händelsen påverkar inte personens normala liv.

Moderat: Händelsen orsakar försämring av funktion men påverkar inte hälsan. Händelsen ger obehag och/eller besvär/hinder.

Svår: Händelsen orsakar försämring av funktion eller arbetsförmåga eller utgör en hälsorisk för personen.

16.2.2 Bedömning av samband

Sannolik: Klinisk händelse, inklusive onormala laboratorieanalyser, som uppträder inom rimlig tid efter administrering av interventionen/studieprodukten. Osannolikt att händelsen kan hänföras till underliggande sjukdom, andra läkemedel eller annan behandling.

Möjlig: Klinisk händelse, inklusive laboratorieanalyser, som uppträder inom rimlig tid efter administrering av interventionen/studieprodukten. Händelsen kan förklaras av underliggande sjukdom eller andra läkemedel.

Osannolik: Klinisk händelse, inklusive onormala laboratorieanalyser, som tillfälligt kan relateras till administrering av interventionen/studieprodukten. Händelsen är osannolikt relaterad till interventionen/studieprodukten och kan förklaras av andra läkemedel eller underliggande sjukdom.

Icke möjlig att klassificera: Händelsen kan inte klassificeras på grund av att information saknas eller att händelsen inte är verifierad.

17 RAPPORTERING AV OÖNSKADE MEDICINSKA HÄNDELSER

17.1 RAPPORTERING AV OÖNSKADE MEDICINSKA HÄNDELSER (AE)

Alla oönskade händelser som sker i direkt anslutning till SERALB-125 dosen skall betraktas som en AE och dokumenteras i enlighet med ICH-GCP principerna.

17.2 ALLVARLIGA OÖNSKADE MEDICINSKA HÄNDELSER (SAE)

Allvarliga oönskade medicinska händelser ska rapporteras till sponsor på ett särskilt SAE-formulär inom 24 timmar från att prövaren fått kännedom om SAE:n. Uppföljande information som beskriver utfall och hantering av SAE:n ska rapporteras så fort den informationen finns tillgänglig. Originalet ska sättas in i CRF.

17.3 RAPPORTERING AV MISSTÄNKTA, OFÖRUTSEDDA, ALLVARLIGA OÖNSKADE REAKTIONER (SUSAR)

FINAL

Sponsor ska ansvara för att all relevant information om misstänkta, oförutsedda, allvarliga oönskade reaktioner registreras och rapporteras till EudraVigilans och till berörda etikprövningsnämnder. SUSARs som är dödliga eller livshotande skall rapporteras till EudraVigilans snarast möjligt och senast inom 7 dagar efter att det inträffade har blivit känt hos sponsor. Relevanta uppföljande uppgifter ska därefter sändas in inom ytterligare 8 dagar. Övriga SUSARs rapporteras snarast möjligt och senast inom 15 dagar efter det att de kommit till sponsors kännedom. För denna typ av rapportering har sponsor kontrakterat Karolinska Trial Alliance, som kommer att ansvara för att detta sker.

17.4 UPPFÖLJNING AV OÖNSKADE MEDICINSKA HÄNDELSER

Ansvarig prövare (läkare) kommer att vara närvarande eller omedelbart tillgänglig under hela försöket och kommer att göra bedömning av svårighetsgrad ifall oönskad medicinsk händelse uppstår. Oönskade medicinska händelser som inte relaterar till studien följs upp inom ramen för det kliniska arbetet och dess rutiner.

18 STATISTIK OCH DATAHANTERING

18.1 DATAHANTERING

Alla laboratorievärden kommer att tryckas ut från patientjournal (Take Care) avidentifieras och bifogas till CRF. Alla värden av vitala parametrar under och efter injektionen dokumenteras direkt på en kodad anestesijournal av sköterska eller av prövare. Denna journal blir en del av CRF. Vid operationens slut skall den övriga anestesijournalen kopieras och avidentifieras och bifogas till CRF. Övriga dokument som sparas i CRF efter kodning och avidentifiering är: pre-anestesibedömning, journal, läkemedelslista från TC, operationsberättelse och övervakningsjournaler fram till 3 dagar efter den andra mätomgången med isotoper.

Data från masspektrometerns mätningar av fenylalanin exporteras som ett dataark via ett USB-minne.

Samtliga data som berör end-points kommer att läggas i en access-databas som låses när datainsamlingen är komplett.

Avidentifierade och kodade demografiska patientdata preoperativt, per-operativt och postoperativt kommer att sammanställas och läggas i access-databas.

18.2 STATISTISK ANALYS

Det finns stöd för att synteshastigheten för albumin, mätt som fraktionell eller absolut synteshastighet, ökar av den generella inflammationen som uppstår efter denna kirurgi. Vidare att kapillärläckaget för albumin ökar mätt som fraktion av albumin i blodet respektive som totalt utflöde av albumin från blodet. Endpoint är förändringen i dessa fyra variabler. För att ta steget från fraktioner till absoluta värden krävs också plasmavolym, P-albumin och kroppsvikt. All data kommer att presenteras som medelvärde med standardavvikelse (SD), alternativt som median och range om data ej skulle visa sig normalfördelade. Ändringarna i variablerna kommer att testas med 2-sidigt parat t-test alternativt Wilcoxon med signifikansnivå 0,05.

Vidare kommer de inbördes korrelationerna mellan de fyra kinetiska albuminvariablerna ovan liksom med P-albumin att beräknas och presenteras med korrelationskoefficienter. Att ändringar i respektive kapillärläckage och syntes skulle kunna korrelera är rimligt, men ej tidigare beskrivet.

Samband mellan ovanstående kinetiska albuminvariabler och graden av SIRS (HR, LPK, temperatur, andningsfrekvens) inklusive CRP kommer att exploreras. Det är rimligt att tro att graden av inflammation påverkar ändringen av albuminvariablerna. Påverkan av pre-operativ viktförlust på albuminets pre-operativa kinetiska variabler kommer också att analyseras, eftersom viktförlust skulle kunna vara en faktor som redan pre-operativt påverkar albuminets kinetiska variabler. Korrelationer kommer att redovisas som Pearson´s korrelationskoefficient r eller Spearman´s rank correlation r_s beroende på datas karaktär.

FINAL

Demografiska pre-operativa data som t ex ålder, längd, vikt, BMI, kön, viss rutin lab, preoperativ viktförlust, varaktighet av pancreassjukdom, diagnoser, aktuella läkemedel, genomgången cellgiftsbehandling och ASA-klass kommer att presenteras som antal, medelvärden och standardavvikelse, eller median range beroende på datas natur. Likaledes redovisas per- och postoperativ demografi t ex operationsblödning, tillförda anestesiläkemedel inklusive epiduralanestesi (doser, mängd), givna vätskeinfusioner inklusive blodprodukter (mL), utförd operation, post-operativa medicinska eller kirurgiska komplikationer, post-operativ läkemedelstillförsel inklusive epidural, och labprover enligt rutinprovtagning.

18.3 BESTÄMNING AV ANTAL FÖRSÖKSPERSONER

Under punkt 1 i detta protokoll anger vi det totala antalet patienter till 16. Det högre antalet är ett sätt att ta höjd för eventuella bortfall, men undersökningarna är så pass kostsamma att avsikten är att avbryta rekryteringen när vi nått 10 evaluerbara patienter.

Med 10 evaluerbara patienter blir power 80 % att upptäcka en skillnad med effektstorlek 1 med ett 2-sidigt t-test och en signifikansnivå på 5 %. Den absoluta albuminsynteshastigheten har uppmätts till 131 ± 21 mg/kg/dag hos friska försökspersoner (kontrollgrupp) där intraindividuella standarddeviationen vid upprepad mätning efter 2,5 timmar var 11 mg/kg/dag [Barle 2002] vilket kan uppfattas som metodens osäkerhet i den specifika och mycket kontrollerade situationen. Vid upprepad mätning på IVA-patienter med 5 dagars mellanrum så var motsvarande värden 233 ± 67 respektive 70 mg/kg/dag [Barle 2001a]. Det finns anledning att tro att vi i detta fall hamnar mellan dessa siffror. Om spridningen i ändringen är 30 kan alltså en skillnad i absolut synteshastighet på 30 mg/kg/d detekteras med 80 % power vilket vi bedömer som en kliniskt relevant förändring.

Ett antal patienter kan förväntas ha förhöjt kapillärläckage (TER) i den basala mätningen (före operationen) pga hypertoni, diabetes och malignitet, men detta hanteras med att patienterna är sina egna kontroller.

Med 10 patienter finns det 80% power att upptäcka en korrelation mellan t ex kapillärläckage och synteshastighet om r är större än 0,71 ($r^2 \geq 0.5$) med ett två-sidigt test och en signifikansnivå på 5%.

Vi har inte funnit några publicerade mätningar av varken synteshastighet eller kapillärläckage för albumin i den aktuella patientgruppen. Antaganden om fördelningar och ändringar blir därmed osäkra. Det finns en risk för att denna pilotstudie visar sig ha lite för svag power, men det är mycket resurskrävande mätningar, och vi får i så fall inkomma med ett amendment att få utöka undersökningen. Det stora antalet korrelationer ovan får ses mot bakgrund av att detta är en explorativ pilotstudie av fysiologiska förändringar vid planerad kirurgi. Det finns därmed en avsikt att studien ska kunna vara hypotesgenererande.

FINAL

19 TILLGÅNG TILL KÄLLDATA

Prövaren ansvarar för att den journalansvarige på kliniken upprättar sekretessförbindelse mellan monitor eller annan oberoende granskare (t.ex. auditör) som ges tillåtelse att verifiera data i patientjournalen. För detta krävs patientens skriftliga samtycke.

20 KVALITETSKONTROLL

20.1 MONITORERING

Prövaren med personal ska avsätta tid för monitorering, audits, myndighetsinspektioner. Sponsorn ansvarar för att monitorering görs av oberoende monitor med kunskaper i GCP, för att säkerställa att

studien utförs enligt GCP, studieprotokollet och myndighetskrav. Sponsorn har kontrakterat Karolinska Trial Alliance för monitorering i denna studie.

21 ETIK

21.1 ETIKPRÖVNINGSNÄMNDEN

Prövaren ansvarar för ansökan om godkännande från etikprövningsnämnden. Sponsorns ansvar är att skicka kopia på godkännandet till läkemedelsverket.

.

21.2 ETISKT GENOMFÖRANDE AV STUDIEN

Studien kommer att utföras enligt studieprotokollet, GCP, myndighetskrav och Helsingfors-deklarationen.

22 DATAHANTERING OCH ARKIVERING

22.1 CASE REPORT FORMS

Data som samlas in i studien i CRF kommer att överföras till en databas som sponsorn ansvarar för.

Data som samlas in i studien kommer att registreras i så kallade Case Report Forms (CRF) där patienten endast är identifierad med ett kodnummer samt initialer. Kodnyckeln förvaras hos prövaren på sådant sätt att obehöriga inte kan ta del av den. Avidentifierade och kodnumrerade kopior av journalhandlingar såsom anestesijournal, övervakningsblad för postoperativ vård, journaltext (Take Care) och lab-listor (Take Care) kommer också att ingå i patientens CRF.

22.2 ARKIVERING

Studiedokumenten kommer att arkiveras i minst 10 år efter att studierapporten har inkommit till läkemedelsverket. Dokumenten ska arkiveras i läsbart skick för ev. framtida audit eller myndighetsinspektion.

23 FINANSIERING OCH FÖRSÄKRING

Studien finansieras med forskningsanslag. Patienterna är försäkrade via läkemedelsförsäkringen och patientskadeförsäkringen.

24 PUBLICERING AV RESULTAT

Avsikten är att publicera resultatet i en vetenskaplig tidskrift.

FINAL

25 REFERENSER

Ballmer PE, McNurlan MA, Milne E, et al. (1990). Measurement of albumin synthesis in humans: a new approach employing stable isotopes. Am J Physiol E797-803

Ballmer PE, Ochsenbein AF, Schütz-Hofmann S. (1994). Transcapillary escape rate of albumin positively correlates with plasma albumin concentration i acute but not in chronic inflammatory disease. Metabolism 43: 697-705

Barle H, Gamrin L, Essén P, et al. (2001a). Growth hormone does not affect albumin synthesis in the critically ill. Intensive Care Med 27: 836-43

Barle H, Råhlén L, Essén P, et al. (2001b). Stimulation of human albumin synthesis and gene expression by growth hormone threatment. Clin Nutr 20: 59-67

Barle H, Januszkiewicz A, Hållström L, et al. (2002). Albumin synthesis in humans increases immediately following the administration of endotoxin. Clin Sci 103: 525-31

Barle H, Hammarqvist F, Westman B, et al. (2006). Synthesis rates of total liver protein and albumin are both increased in patients with an acute inflammatory response Clin Sci 110, 93–9

Cochrane Injuries Study Group. (1998). Human albumin administration in critically ill patients: systematic review of randomised controlled trials. BMJ 317: 235-40

Cochrane Database Syst Rev (2004). Human albumin solution for resuscitation and volume expansion in critically ill patients. Okt 18 (4): CD001208.

Dubois M-J, Crellana-Jimenez C, Melot C, et al. (2006). Albumin administration improves organ function in critically ill hypoalbuminemic patients: a prospective, randomized, controller pilot study. Crit Care Med 34: 2536-40

Essen P, McNurlan MA, Gamrin L, et al. (1998). Tissue protein synthesis rates in critically ill patients. Crit Care Med 26: 92-100

Fleck A, Hawker F, Wallace PI, et al. (1985) Increased vascular permeability: a major cause of hypoalbuminaemia in disease and injury. Lancet 1: 781-4

Koot BGP, Houwen R, Pot D-J, et.al. (2004). Congenital analbuminaemia: biochemical and clinical implications. A case report and literature review. Eur J Pediatr 163: 664-70

Moshage HJ, Janssen JAM, Franssen JH, et al. (1987) Study of the molecular mechanism of decreased liver synthesis of albumin in inflammation. J Clin Invest 79: 1635-41

SAFE study investigators (2004). A comparison of albumin and saline for fluid resuscitation in the intensive care unit. New Eng J Med 350: 2247-56

van Eijk LTGJ, Pickkers P, Smits P, et al. (2005), Microvascular permeability during experimental human endotoxemia: an open intervention study. Crit Care 2005, 9: R157-64

Vincent JL. (2009) Relevance of albumin in modern critical care medicine. Best Pract Res Clin Anaesthesiol. 23:183-91

FINAL

26 SIGNATUR

En signatursida per studiecenter ska signeras av sponsor, koordinerande prövare och ansvarig prövare. Ange vad signaturen innebär (att personen har läst och förstått protokollet och håller med om dess innehåll).

Ansvarig prövare
Åke Norberg
Anestesi och intensivvårdskliniken
Karolinska Universitetssjukhuset Huddinge

Signatur | Datum

Sponsor
Åke Norberg
Anestesi och intensivvårdskliniken
Karolinska Universitetssjukhuset Huddinge

Signatur | Datum

Annan function
Namn, titel

Signatur | Datum

FINAL

27 BILAGOR

27.1 Flödesschema

		DAG - 14 (DAG -28 TILL -2)	DAG -1 (DAG -3 TILL -1)	DAG 0	DAG 2 (DAG 1 TILL DAG 4)	DAG 5 (DAG 4 TILL DAG 7)							
Läkarbesök		X	X	X	X	X							
Provtagning RUTIN		X		X	X	X							
Muntlig information om studien		X	X										
Skriflig patientinformation		X											
Skrifligt samtycke		(X)	X										
Inklusion/exklusionskriterier			X										
Demografiska data			X	X	X	X							
Aktuella sjukdomar			X		X	X							
Aktuell medicinering			X		X	X							
Blodprovtagning: P-Albumin, P-CRP				X	X								
Blodprovtagning: B-LPK				X									
Urin-HCG (kvinnor FERTILA)			**X**										
Radiojod injektion första dos				X									
Radiojod injektion andra dos					X								
Isotopmärkt injektion m fenylalanin				X	X								
Vitala parametrar (BT, AF, puls, temp)			X	X	X	X							
Adverse events				X	X								

FINAL
