# Supplementary material for: Albumin Kinetics in Patients Undergoing Major Abdominal Surgery
Source: PLoS One. 2015 Aug 27;10(8):e0136371. doi: 10.1371/journal.pone.0136371 (PMC4552033; doi:10.1371/journal.pone.0136371)
Supplement: S1 Table — Data for all individual subjects in Tables 1–3, extra anthropometry, mass balance and fluid balance. (PDF) [file pone.0136371.s004.pdf]

Table 1.
Patient charachteristics, individual data

subject	**# 1**	**# 2**	**# 3**	**# 4**	**# 5**	**# 6**	**# 7**	**# 8**	**# 9**	**# 10**	**mean**	**SD**	**median**	**range**
gender	m	f	f	m	f	m	f	m	m	m				m:6, f:4
age, year	52	55	63	64	44	65	65	67	69	66	61	8	65	
height, cm	183	164	159	179	168	189	160	173	173	179	173	10	173	
weight, kg	80	68	66	83	62	83	51	65	86	86	73	12	74	
BMI, kg/cm2	23,9	25,3	26,1	25,9	22,0	23,2	19,9	21,7	28,7	26,8	24,4	2,7	24,6	
weight loss, kg	0	0	0	0	0	0	0	0	5	0	na	na	0	0-5
P-cratinine, µmol/L	46	49	50	98	66	89	47	70	81	74	67	19	68	
P-bilirubin	7	9	5	8	4	9	7	22	3	7	8	5	7	
P-ASAT	0,43	0,66	0,28	0,62	0,4	0,35	0,25	0,59	0,28	0,43	0,43	0,15	0,42	
P-ALAT	0,94	1,02	0,21	0,63	0,47	0,18	0,25	0,65	0,21	0,33	0,49	0,31	0,40	
P-GT	0,44	1,6	0,47	0,87	0,69	0,67	0,7	2,4	0,41	0,48	0,87	0,64	0,68	
P-pancreasamylas	0,34	0,13	1,51	0,2	0,13	1,67	0,13	0,16	0,84	0,49	0,56	0,59	0,27	
ASA	2	3	2	3	2	2	3	3	3	3				2: 4, 3: 6

Table 2.
Physiological data before and 2 days after major pancreatic surgery, individual values

**Day 0**	**subject**	**# 1**	**# 2**	**# 3**	**# 4**	**# 5**	**# 6**	**# 7**	**# 8**	**# 9**	**# 10**	**mean**	**SD**	**median**	**range**
SAP	mmHg	115	126	88	131	140	127	125	112	125	150	124	17	126	
DAP	mmHg	60	64	47	65	70	63	45	58	65	75	61	9	64	
MAP	mmHg	78	85	61	87	93	84	72	76	85	100	82	11	85	
Heart Rate	beats/min	68	77	53	65	91	51	62	68	64	75	67	12	67	
Respiratory Rate	per min	12	18	14	11	14	11	14	12	19	12	13,7	2,8	13	11-19
Body temperature	°C	36,3	36,9	36,8	36,3	37,4	36,4		36,5	36,7	36,4	36,6	0,4	36,5	
B-WBC	10^9/L	6,8	3,2	7	5,8	7,2	5,4	5,9	7,5	5	5,6	5,9	1,3	5,9	
P-CRP	mg/L	0	3	1	7	3	1	1	1	16	1	3	5	1	0,47-16,17
B-Hb	g/L	131	141	149	138	147	149	135	134	98	153	138	16	140	
Body weight	kg	81,2	68	64,8	82,7	62,5	83,4	50,6	65,1	85,7	85	72,9	12,2	74,6	

**Day 2**	**subject**	**# 1**	**# 2**	**# 3**	**# 4**	**# 5**	**# 6**	**# 7**	**# 8**	**# 9**	**# 10**	**mean**	**SD**	**median**	**range**
SAP	mmHg	145	124	108	171	121	126	160	113	93	132	129	24	125	
DAP	mmHg	65	54	55	91	62	75	60	69	66	62	66	11	64	
MAP	mmHg	92	77	73	118	82	92	93	84	75	85	87	13	85	
Heart Rate	beats/min	71	95	90	102	94	61	88	96	75	68	84	14	89	
Respiratory Rate	per min	17	20	19	15	18	16	15	15	18	15	16,8	1,9	16,5	15-20
Body temperature	°C	37,2	38,8	38	38,1	37,8	37,2	38	37,6	37,4	38,1	37,8	0,5	37,9	
B-WBC	10^9/L	7	9	10,1	21	11,8	15,1	10,8	8	19,3	12,4	12,5	4,7	11,3	
P-CRP	mg/L	42	135	145	234	120	36	112	25	195	200	124	73	127	25-234
B-Hb	g/L	104	92	98	113	95	115	104	96	102	114	103	8	103	
Body weight	kg	80,3	73,3	68,9	86,6	65,7	83,8	52	68,7	89,7	87,6	75,7	12,0	76,8	

Table 2. continued

Day 2 -0	subject	# 1	# 2	# 3	# 4	# 5	# 6	# 7	# 8	# 9	# 10	mean	SD	median	range
SAP	mmHg	30	-2	20	40	-19	-1	35	1	-32	-18	5	25	0	
DAP	mmHg	5	-10	8	26	-8	12	15	11	1	-13	5	12	7	
MAP	mmHg	13,33333	-7,33333	12	30,66667	-11,6667	7,666667	21,66667	7,666667	-10	-14,6667	5	15	8	
Heart Rate	beats/min	3	18	37	37	3	10	26	28	11	-7	17	15	15	
Respiratory Rate	per min	5	2	5	4	4	5	1	3	-1	3	3	2	4	-1 - 5
Body temperature	°C	0,9	1,9	1,2	1,8	0,4	0,8		1,1	0,7	1,7	1	1	1	
B-WBC	10^9/L	0,2	5,8	3,1	15,2	4,6	9,7	4,9	0,5	14,3	6,8	7	5	5	
P-CRP	mg/L	41,62	131,6	144,08	227,04	116,98	34,89	110,89	24,1	178,45	198,88	121	70	124	24-227
B-Hb	g/L	-27	-49	-51	-25	-52	-34	-31	-38	4	-39	-34	17	-36	
Body weight	kg	-0,9	5,3	4,1	3,9	3,2	0,4	1,4	3,6	4	2,6	3	2	3	

Table 3.
Albumin kinetic parameters, individual data

Day 0	subject	# 1	# 2	# 3	# 4	# 5	# 6	# 7	# 8	# 9	# 10	mean	SD	median
P-alb	g/L	35,3	28,8	36,7	39,3	35,6	32,2	32,6	33,4	26,7	34,7	33,5	3,7	34,1
Plasma volume	mL	4197	2924	2721	3078	2644	3938	2397	3414	4178	3614	3311	655	3246
Blood volume	L	6,40	4,69	4,25	4,86	4,14	6,60	3,73	5,28	5,46	6,19	5,16	1,00	5,07
IAM	g	148	84	100	121	94	127	78	114	112	125	110	21	113
FSR	%/d	8,0	10,1	10,6	9,2	12,0	11,1	9,1	14,6	21,5	10,4	11,7	3,9	10,5
ASR	mg/kg/d	147	125	164	135	180	169	141	255	280	154	175	52	159
TER	%/h	3,2	5,0	2,5	0,7	7,7	4,1	9,9	5,4	5,7	5,0	4,9	2,6	5,0
Alb mass flow	g/h	4,8	4,2	2,5	0,8	7,3	5,2	7,7	6,2	6,3	6,2	5,1	2,1	5,7
Body weight	kg	81,2	68	64,8	82,7	62,5	83,4	50,6	65,1	85,7	85	72,9	12,2	74,6
Haematocrit	fraction	0,403	0,433	0,418	0,424	0,4195	0,457	0,415	0,412	0,304	0,469	0,415	0,044	0,419

Day 2	subject	# 1	# 2	# 3	# 4	# 5	# 6	# 7	# 8	# 9	# 10	mean	SD	median
P-alb	g/L	24,9	17,7	25,5	25,6	19,5	23,9	23,1	19	18,2	23,4	22,1	3,1	23,3
Plasma volume	mL	4419	2629	2845	2795	3389	3686	2791	3917	3341	3347	3316	574	3344
Blood volume	L	5,93	3,35	3,72	3,91	4,37	5,20	3,75	5,08	4,44	4,70	4,45	0,80	4,41
IAM	g	110	47	73	72	66	88	64	74	61	78	73	17	72
FSR	%/d	13,1	7,9	13,6	11,3	12,8	20,3	13,8	23,8	19,0	14,6	15,0	4,7	13,7
ASR	mg/kg/d	180	50	143	93	128	213	171	258	129	131	150	59	137
TER	%/h	2,5	4,7	4,7	10,0	7,0	4,8	3,9	3,2	7,7	6,8	5,5	2,3	4,8
Alb mass flow	g/h	2,8	2,2	3,4	7,2	4,6	4,2	2,5	2,4	4,7	5,3	3,9	1,6	3,8
Body weight	kg	80,3	73,3	68,9	86,6	65,7	83,8	52	68,7	89,7	87,6	75,7	12,0	76,8
Haematocrit	fraction	0,322	0,286	0,304	0,349	0,295	0,355	0,322	0,298	0,316	0,352	0,320	0,025	0,319

Day 2 - day 0	subject	# 1	# 2	# 3	# 4	# 5	# 6	# 7	# 8	# 9	# 10	mean	SD	median
P-alb	g/L	-10,4	-11,1	-11,2	-13,7	-16,1	-8,3	-9,5	-14,4	-8,5	-11,3	-11,5	2,6	-11,2
Plasma volume	L	222	-294	124	-283	745	-252	394	503	-837	-267	5	475	-64
Blood volume	L	-0,47	-1,34	-0,53	-0,96	0,23	-1,40	0,02	-0,21	-1,02	-1,49	-0,72	0,61	-0,75
IAM	g	-38	-38	-27	-49	-28	-39	-14	-40	-51	-47	-37	11	-38
FSR	%/d	5,1	-2,1	3,0	2,1	0,8	9,1	4,7	9,2	-2,5	4,2	3,4	4,0	3,6
ASR	mg/kg/d	33	-74	-20	-41	-52	43	30	3	-152	-23	-25	59	-22
TER	%/h	-0,7	-0,3	2,2	9,3	-0,7	0,7	-6,0	-2,2	2,1	1,8	0,6	3,9	0,2
Alb mass flow	g/h	-2,0	-2,0	0,9	6,3	-2,7	-1,0	-5,2	-3,8	-1,6	-0,9	-1,2	3,1	-1,8

Extra anthropometric data

Data day 0 / Subject	101	102	103	104	105	106	107	108	109	110	Mean	SD	Median
Blood pressure before dose, DAP mmHg	115	126	88	131	140	127	125	112	125	150	**123,9**	**16,7**	**125,5**
Blood pressure before dose, SAP mmHg	60	64	47	65	70	63	45	58	65	75	**61,2**	**9,3**	**63,5**
Heart rate before dose	68	77	53	65	91	51	62	68	64	75	**67,4**	**11,7**	**66,5**
Respiratory rate before dose	12	18	14	11	14	11	14	12	19	12	**13,7**	**2,8**	**13**
Body temperature before dose	36,3	36,9	36,8	36,3	37,4	36,4	miss	36,5	36,7	36,4	**36,6**	**0,4**	**36,5**
B-LPK	6,8	3,2	7	5,8	7,2	5,4	5,9	7,5	5	5,6	**5,9**	**1,3**	**5,85**
P-CRP	0,47	3,38	0,52	6,51	2,9	0,83	0,63	0,97	16,17	0,68	**3,3**	**4,9**	**0,9**
P-Albumin	35,3	28,8	36,7	39,3	35,6	32,2	32,6	33,4	26,7	34,7	**33,5**	**3,7**	**34,05**
P-CRP routine d-30 till d0	2	4	0,9	7	3	2	0,9	1	30	0,9	**5,2**	**8,9**	**2**
P-Albumin routine d-30 till d0	38	35	44	40	41	37	37	34	28	miss	**37,1**	**4,6**	**37**
B-Hb routine d-30 till d0	131	141	149	138	147	149	135	134	98	153	**137,5**	**15,7**	**139,5**
P-kreatinin	46	64	50	99	64	89	44	70	81	76	**68,3**	**18,4**	**67**
P-bilirubin	7	9	5	8	4	9	7	22	3	7	**8,1**	**5,3**	**7**
P-ASAT	0,43	0,66	0,28	0,62	0,4	0,35	0,25	0,59	0,28	0,43	**0,4**	**0,1**	**0,415**
P-ALAT	0,94	1,02	0,21	0,63	0,47	0,18	0,25	0,65	0,21	0,33	**0,5**	**0,3**	**0,4**
P-GT	0,44	1,6	0,47	0,87	0,69	0,67	0,7	2,4	0,41	0,48	**0,9**	**0,6**	**0,68**
P-pancreasamylas	0,34	<0,13	1,51	0,2	<0,13	1,67	<0,13	0,16	0,84	0,49	**0,7**	**0,6**	**0,49**
Body Weight x	81,2	68	64,8	82,7	62,5	83,4	50,6	65,1	85,7	85	**72,9**	**12,2**	**74,6**
Operating time, min	340	230	522	501	365	300	208	598	359	357	**378,0**	**126,3**	**358**
Op-bleeding, ml	3700	2600	800	1800	700	650	500	1400	850	520	**1352**	**1063**	**825**

Data dag 2	101	102	103	104	105	106	107	108	109	110	Mean	SD	Median
Blood pressure before dose, DAP mmHg	145	124	108	171	121	126	160	113	93	132	**129,3**	**23,8**	**125**
Blood pressure before dose, SAP mmHg	65	54	55	91	62	75	60	69	66	62	**65,9**	**10,8**	**63,5**
Heart rate before dose	71	95	90	102	94	61	88	96	75	68	**84,0**	**14,0**	**89**
Respiratory rate before dose	17	20	19	15	18	16	15	15	18	15	**16,8**	**1,9**	**16,5**
Body temperature before dose	37,2	38,8	38	38,1	37,8	37,2	38	37,6	37,4	38,1	**37,8**	**0,5**	**37,9**
B-LPK	7	9	10,1	21	11,8	15,1	10,8	8	19,3	12,4	**12,5**	**4,7**	**11,3**
P-CRP	42,09	134,98	144,6	233,55	119,88	35,72	111,52	25,07	194,62	199,56	**124,2**	**72,8**	**127,43**
P-Albumin	24,9	17,7	25,5	25,6	19,5	23,9	23,1	19	18,2	23,4	**22,1**	**3,1**	**23,25**
P-CRP routine d2	46	125	131	250	116	32	110	46	174	177	**120,7**	**68,3**	**120,5**
P-albumin routine d2	24	16	25	28	20	23	23	18	18	22	**21,7**	**3,7**	**22,5**

Extra anthropometric data, continued

Data dag 2	101	102	103	104	105	106	107	108	109	110	Mean	SD	Median
B-Hb routine d2	104	92	98	113	95	115	104	96	102	114	**103,3**	**8,3**	**103**
P-kreatinin routine d2	41	54	36	153	54	72	33	67	82	75	**66,7**	**34,7**	**60,5**
P-bilirubin routine d2	13	5	6	13	4	8	10	11	7	8	**8,5**	**3,2**	**8**
P-ASAT routine d2	0,8	0,75	0,61	2,01	0,7	0,71	0,59	10,42	0,68	0,74	**1,8**	**3,1**	**0,725**
P-ALAT routine d2	1,69	0,68	0,62	1,73	0,51	0,43	0,39	6,12	0,32	0,83	**1,3**	**1,8**	**0,65**
P-GT routine d2	0,28	0,53	0,62	0,47	0,28	0,34	0,56	0,61	0,41	0,29	**0,4**	**0,1**	**0,44**
P-pancreasamylas routine d2	0,15	0,66	1,39	3,16	0,18	2,12	0,13	0,2	3,15	3,48	**1,5**	**1,4**	**1,025**
Vikt d2	80,3	73,3	68,9	86,6	65,7	83,8	52	68,7	89,7	87,6	**75,7**	**12,0**	**76,8**
Vikt d2 - vikt x d0	-0,9	5,3	4,1	3,9	3,2	0,4	1,4	3,6	4	2,6	**2,8**	**1,9**	**3,4**
EDA, dos ml/h	10	8	8	13	15	12	15	8	9	6	**10,4**	**3,2**	**9,5**
NRS	2	2	2	3	5	3	3	2	2	2	**2,6**	**1,0**	**2**

STUDIENUMMER	101	102	103	104	105	106	107	108	109	110	Mean	SD	Median
Norepinephrine h post surgery	11	36	12	0	0	0	23	0	17	17	**11,6**	**12,1**	**11,5**
P-CRP routine d0 - d-30	2	4	<1	7	3	2	0,9	1	30	0,9	**5,6**	**9,3**	**2**
P-CRP routine d1	24	53	33	86	62	32	missing	26	86	54	**50,7**	**24,0**	**53**
P-CRP routine d2	46	129	131	250	116	32	110	46	174	177	**121,1**	**68,4**	**122,5**
P-CRP routine d3	21	83	186	276	86	61	90	45	missing	257	**122,8**	**93,4**	**86**
P-CRP routine d4	10	38	134	232	44	missing	53	23	missing	180	**89,3**	**82,2**	**48,5**
P-CRP routine d5	21	68	103	252	43	missing	50	missing	missing	missing	**89,5**	**84,2**	**59**
P-CRP routine d6	10	97								64			
LPK routine d1	7,4	8,3			12,1	17,9					**11,4**	**4,8**	**10,2**
B-LPK (study assessment)	7	9	10,1	21	11,8	15,1	10,8	8	19,3	12,4	**12,5**	**4,7**	**11,3**
LPK routine d3	5	10,6	10,4				11,7				**9,4**	**3,0**	**10,5**
LPK routine d4	4,5	9					10,6				**8,0**	**3,2**	**9**
LPK routine d5	5	8,9					9,6				**7,8**	**2,5**	**8,9**

Subject	101	102	103	104	105	106	107	108	109	110	Medel	SD	Median

Mass balance calculations for Hb and albumin

Data 0-60 min

subject	101	102	103	104	105	106	107	108	109	110	Mean	SD	Median
**TER day0**	3,223	5,005	2,484	0,702	7,726	4,085	9,908	5,395	5,676	4,975	**4,918**	**2,601**	**4,990**
**TER day2**	2,534	4,704	4,723	10,046	6,988	4,802	3,888	3,165	7,738	6,808	**5,540**	**2,313**	**4,763**
**PV day0**	4197	2924	2721	3078	2644	3938	2397	3414	4178	3614	**3311**	**655**	**3246**
**PV day2**	4419	2630	2845	2795	3390	3686	2791	3917	3341	3347	**3316**	**574**	**3344**
**CRP day0**	0,47	3,38	0,52	6,51	2,9	0,83	0,63	0,97	16,17	0,68	**3,31**	**4,91**	**0,90**
**CRP day2**	42,1	135,0	144,6	233,6	119,9	35,7	111,5	25,1	194,6	199,6	**124,2**	**72,8**	**127,4**
**P-alb day0**	35,3	28,8	36,7	39,3	35,6	32,2	32,6	33,4	26,7	34,7	**33,5**	**3,7**	**34,1**
**P-alb day2**	24,9	17,7	25,5	25,6	19,5	23,9	23,1	19	18,2	23,4	**22,1**	**3,1**	**23,3**
**IAM day0**	148	84	100	121	94	127	78	114	112	125	**110**	**21**	**113**
**IAM day2**	110	47	73	72	66	88	64	74	61	78	**73**	**17**	**72**
**Jalb day0**	477,5	421,5	248,0	84,9	727,3	518,1	774,3	615,2	633,1	624,0	**512,4**	**214,6**	**566,7**
**Jalb day2**	278,8	218,9	342,7	718,9	461,9	423,1	250,7	235,6	470,5	533,2	**393,4**	**159,3**	**382,9**
**diff TER**	-68,9	-30,1	224,0	934,4	-73,8	71,7	-602,0	-223,0	206,3	183,3	**62,2**	**392,9**	**20,8**
**diff PV**	222	-294	124	-283	745	-252	394	503	-837	-267	**5**	**475**	**-64**
**diff CRP**	41,6	131,6	144,1	227,0	117,0	34,9	110,9	24,1	178,5	198,9	**120,9**	**70,3**	**124,3**
**diff Palb**	-10,4	-11,1	-11,2	-13,7	-16,1	-8,3	-9,5	-14,4	-8,5	-11,3	**-11,5**	**2,6**	**-11,2**
**diff IAM**	-38	-38	-27	-49	-28	-39	-14	-40	-51	-47	**-37**	**11**	**-38**
**diff Jalb**	-198,7	-202,5	94,7	634,0	-265,4	-95,0	-523,6	-379,7	-162,6	-90,8	**-119,0**	**313,1**	**-180,6**

subject	101	102	103	104	105	106	107	108	109	110	Mean	SD	Median
**Bleeding, ml**	3700	2600	800	1800	700	650	500	1400	850	520	**1352**	**1063**	**825**
**E-konc, ml**	1500	1000	0	0	0	0	500	500	750	0	**425**	**528**	**250**
**Drains, ml**	825	240	685	640	590	505	260	445	1050	600	**584**	**244**	**595**
**Plasma, ml**	1500	250	0	0	0	0	500	250	0	0	**250**	**471**	**0**
**Albumin [g]**	0	0	25	37,5	0	0	0	0	0	0	**6,25**	**13,50**	**0,00**
**Hb day0**	131,0	141,0	136,0	138,0	136,5	149,0	135,0	134,0	98,0	153,0	**135,2**	**14,7**	**136,3**
**Mean Hb (per-op)**	101,0	105,6	122,0	108,0	109,3	146,0	100,7	120,1	94,2	127,0	**113,4**	**15,5**	**108,6**
**htk day0**	0,4	0,4	0,4	0,4	0,4	0,5	0,4	0,4	0,3	0,5	**0,4**	**0,0**	**0,4**
**BV day0**	7	5	4	5	4	7	4	5	6	6	**5**	**1**	**5**
**MHb day0**	868	680	597	692	584	1005	520	732	566	965	**721**	**171**	**686**
**Hb day2**	104,0	92,0	98,0	113,0	95,0	115,0	104,0	96,0	102,0	114,0	**103,3**	**8,3**	**103,0**
**htk day2**	0,32	0,29	0,30	0,35	0,30	0,36	0,32	0,30	0,32	0,35	**0,32**	**0,03**	**0,32**
**BV day2**	6,3	3,6	3,9	4,1	4,6	5,4	3,9	5,4	4,7	4,9	**4,7**	**0,8**	**4,7**
**MHb day2**	650	327	385	463	440	626	411	516	478	561	**486**	**104**	**471**
**MB Hb day2**	764,1	590,2	489,4	487,7	498,4	902,1	465,7	557,0	611,2	889,6	**625,6**	**166,9**	**573,6**
**Hb "missing g"**	114,0	263,1	103,9	24,8	58,3	275,9	55,1	41,1	132,9	328,3	**139,8**	**109,6**	**109,0**

Mass balance calculations for Hb and albumin

subject	101	102	103	104	105	106	107	108	109	110	Mean	SD	Median
**Blood "missing" Hb**	1096	2860	1060	220	613	2399	530	428	1303	2880	**1339**	**1012**	**1078**
**IAM losses**	-85,36	-43,79	-25,61	-46,19	-20,69	-18,06	-12,64	-30,62	-27,35	-17,26	**-32,76**	**21,50**	**-26,48**
**IAM gains**	51,00	8,50	25,00	37,50	0,00	0,00	17,00	8,50	0,00	0,00	**14,75**	**17,92**	**8,50**
**IAM day2 MB**	113,8	48,9	99,3	112,3	73,4	108,8	82,5	91,9	84,2	108,1	**92,3**	**20,6**	**95,6**
**alb MB**	34,4	35,3	0,6	8,7	20,7	18,1	-4,4	22,1	27,3	17,3	**18,0**	**13,2**	**19,4**
**alb "missing g"**	4	2	27	41	7	21	18	17	23	30	**19**	**12**	**19**
**Blood "missing" alb**	151	134	1047	1591	377	864	781	921	1286	1275	**843**	**494**	**892**
**BV Nadler day0**	5,43	4,00	3,80	5,38	3,92	5,75	3,33	4,60	5,27	5,48	**4,70**	**0,87**	**4,93**
**BV Nadler day2**	5,44	4,18	3,89	5,50	4,05	5,78	3,36	4,72	5,39	5,53	**4,78**	**0,85**	**5,05**
**PVNadler day0**	3,44	2,43	2,35	3,30	2,43	3,36	2,07	2,87	3,81	3,14	**2,92**	**0,58**	**3,01**
**PVNadler day2**	3,84	3,09	2,82	3,75	2,96	3,91	2,38	3,44	3,84	3,76	**3,38**	**0,54**	**3,59**
**gender**	m	f	f	m	f	m	f	m	m	m			
**height, cm**	183	164	159	179	168	189	160	173	173	179	**173**	**10**	**173**
**weight day0, kg**	80,00	68,00	66,00	83,00	62,00	83,00	51,00	65,00	86,00	86,00	**73,00**	**12,16**	**74,00**
**weight day2, kg**	80,30	73,30	68,90	86,60	65,70	83,80	52,00	68,70	89,70	87,60	**75,66**	**12,05**	**76,80**
**diff weight**	0,30	5,30	2,90	3,60	3,70	0,80	1,00	3,70	3,70	1,60	**2,66**	**1,64**	**3,25**
**diff IAM%**	26	45	27	41	30	31	17	35	45	38	**33**	**9**	**33**
**diff MHb%**	25,12	51,93	35,46	33,08	24,60	37,67	21,04	29,51	15,49	41,81	**31,57**	**10,74**	**31,29**

Abbreviations and explainations

**BV**	Blood volume, L; BV = 0,91 * PV/((1-htk)*1000); an F-ratio of 0.91 is assumed
**BV Nadler**	Anthropometric blood volume according to Nadler 1962, L
**CRP**	Plasma C-reactive protein (sensitive), mikromol/L
**diff**	Difference: day2-day0
**E-konc**	Packed red cells
**Hb**	Blood Haemoglobin, g
**Hb missing**	Mass balance Hb - MHb day2
**Htk**	Blood Hematocrit, fraction
**IAM**	Intravascular albumin mass, g
**IAM day2 MB**	IAM day2 mass balance = IAM day0 - bleeding - losses in drains - occult bleeding + albumin in plasma inf + albumin inf
**Jalb**	Albumin mass flow rate out from the blood g/d
**MB Hb day2**	Mass balance Hb day2 = MHb day0 - bleeding-drains - occult bleeding + red packed cells
**MHb**	Intravascular haemoglobin mass = B-Hb * blood volume
**P-alb**	Plasma albumin, g/L
**Plasma**	Plasma is containing albumin 34 g/L on average
**PV**	Plasma volume, ml
**PV Nadler**	Anthropometric plasma volume derived from BV Nadler * (1 - 0.91 * hkt)
**TER**	Transcapillary escape rate, %/h

Surgery and Fluid Balance

SUBJECT #	101	102	103	104	105	106	107	108	109	110	Mean	sd	Median
**Anaesthesia record**													
Op-bleedning, ml	3700	2600	800	1800	700	650	500	1400	850	520	**1352,0**	**1062,8**	**825**
Diuresis, ml	800	760	1462	787	615	405	550	1100	370	765	**761,4**	**325,6**	**762,5**
Perspiratio, ml	2240	1235	2010	2725	2045	1760	850	2590	1615	2337	**1940,7**	**589,3**	**2027,5**
Drains, ventricel tube, misc, ml	0	50	210	100	0	0	0	200	75	150	**78,5**	**83,7**	**62,5**
Glucose 2,5 mg/ml bufferd per-op, ml	489	900	917	674	396	470	600	660	520	958	**658,4**	**203,0**	**630**
Ringer-Acetat per-op, ml	1427	1000	2109	2460	2070	1505	1300	1728	1800	2069	**1746,8**	**443,4**	**1764**
Volulyte per-op, ml	2855	1900	1782	2500	2732	1330	950	2500	1133	1500	**1918,2**	**692,1**	**1841**
E-konc per-op, ml	1000	1000	0	0	0	0	0	500	500	0	**300,0**	**421,6**	**0**
Plasma per-op, ml	1000	250	0	0	0	0	0	250	0	0	**150,0**	**316,2**	**0**
Albumin 50 mg/ml	0	0	0	250	0	0	0	0	0	0	**25,0**	**79,1**	**0**
Macrodex, ml	0	0	0	0	500	0	0	0	0	0	**50,0**	**158,1**	**0**
Other iv fluids, ml	0	493	640	619	646	50	126	200	500	0	**327,4**	**276,8**	**346,5**
Fluid balance during surgery, ml	-43	798	896	1092	2484	0	1076	1500	1541	914	**1025,8**	**737,8**	**995**
**"Fluid sheet" balance day 1, 06:00**													
Glucose, ml	1850	1800	1880	1500	1581	1900	2400	1800	1500		**1801,2**	**275,4**	**1800**
Ringer-Acetat, ml	3000	3000	3000	4000	2000	3000	2000	3000	2000		**2777,8**	**666,7**	**3000**
Volulyte, ml	3000	2500	2500	2500	2000	1500	1000	2500	2000		**2166,7**	**612,4**	**2500**
E-konc , ml	1000	1000	0	0	0	0	0	500	500	0	**300,0**	**421,6**	**0**
Plasma, ml	1500	250	0	0	0	0	0	250	0	0	**200,0**	**468,4**	**0**
Albumin 50 mg/ml, ml	0	0	500	250	0	0	0	0	0	0	**75,0**	**168,7**	**0**
Macrodex, ml	0	0	0	0	0	0	0	500	0	0	**50,0**	**158,1**	**0**
Fluid for drugs (antibiotics, etc), ml	450	700	600	600	600	684	400	360	950		**593,8**	**180,0**	**600**
Perspiratio, ml			3500	3900	2500		500	3370	2000		**2628,3**	**1254,4**	**2935**
Diuresis, ml	2740	2180	2395	1590	1680	1945	1520	2360	1605	1590	**1960,5**	**431,8**	**1812,5**
Drains, ventricel tube, misc, ml	100	90	340	740	200	275	160	345	300	250	**280,0**	**185,6**	**262,5**
**Take Care day 1, 06:00**													
Glucose, ml	1870	1800	2300	1000	2040	2000	2000	1300	1500	2175	**1798,5**	**410,0**	**1935**
Ringer-Acetat, ml	3000	3000	3000	3000	2000	3000	3000	3000	2000	3000	**2800,0**	**421,6**	**3000**
Volulyte, ml	3000	2500	2500	2500	2000	1500	1000	2500	2000	1500	**2100,0**	**614,6**	**2250**
E-konc , ml	1000	1000	0	0	0	0	0	500	500	0	**300,0**	**421,6**	**0**
Plasma, ml	1500	250	0	0	0	0	0	250	0	0	**200,0**	**468,4**	**0**
Albumin 50 mg/ml, ml	0	0	0	250	0	0	0	0	0	0	**25,0**	**79,1**	**0**
Macrodex, ml	0	0	0	0	500	0	0	500	0	0	**100,0**	**210,8**	**0**
Miscellaneous, ml	350	825	525	626	860	684	384	550	759	380	**594,3**	**187,6**	**588**
**"Fluid sheet" balance day 2, 06:00**													
Diuresis, ml	2870	2635	1760	2260	2230	3350	2055	2055	1500	2030	**2274,5**	**544,1**	**2142,5**
Drains, ml	575	100	225	0	390	230	100	100	750	350	**282,0**	**237,1**	**227,5**

**Take Care day 2, 06:00**													
Glucose, ml	1690	2000	1600	1981	1000	1685	2020	2550	2000	2000	**1852,6**	**400,2**	**1990,5**
Ringer-Acetat, ml	1000	1000	2000	1000	1000	1000	200	1000	1000	1000	**1020,0**	**426,4**	**1000**
Volulyte, ml	0	500	0	0	0	500	500	500	1000	500	**350,0**	**337,5**	**500**
E-konc , ml	500	0	0	0	0	0	500	0	250	0	**125,0**	**212,5**	**0**
Plasma, ml	0	0	0	0	0	0	500	0	0	0	**50,0**	**158,1**	**0**
Albumin 50 mg/ml, ml	0	0	500	500	0	0	0	0	0	0	**100,0**	**210,8**	**0**
Macrodex, ml	0	0	0	0	0	0	0	500	0	0	**50,0**	**158,1**	**0**
Miscellaneous, ml	457	262	529	520	595	527	641	300	200	238	**426,9**	**161,4**	**488,5**
**TC day 2 until sampling**	kl 10:02	kl 09:00	kl 08:20	kl 08:42	kl 09:37	kl 10:03	kl 10:17	kl 08:54	kl 08:38	kl 08:10			
Glucos + chrystalloids, ml	320	240	192	430	0	320	320	230	210	170	**243,2**	**116,0**	**235**
Macrodex + volulyte, ml	0	0	0	0	300	240	0	0	0	0	**54,0**	**114,7**	**0**
**"Fluid sheet" day 2 until sampling**													
Diuresis, ml	790	440	90	130	50	600	115	85	70	140	**251,0**	**262,5**	**122,5**
SUBJECT #	101	102	103	104	105	106	107	108	109	110	Mean	sd	Median
**Sum chrystalloid d0-d2 TC**	8687	9127	10146	8557	7495	9216	8565	8930	7669	8963	**8735,5**	**760,3**	**8808,5**
**Sum colloid d0-d2 TC**	3000	3000	2500	2500	2800	2240	1500	4000	3000	2000	**2654,0**	**680,7**	**2650**
**Sum E-konc TC**	1500	1000	0	0	0	0	500	500	750	0	**425,0**	**527,7**	**250**
**Sum Plasma TC**	1500	250	0	0	0	0	500	250	0	0	**250,0**	**471,4**	**0**
**Sum Albumin TC**	0	0	500	750	0	0	0	0	0	0	**125,0**	**270,0**	**0**
**Sum IN**	14687	13377	13146	11807	10295	11456	11065	13680	11419	10963	**12189,5**	**1430,6**	**11631,5**
**Diuresis d0-d2 "fluid sheets"**	6400	5255	4245	3980	3960	5895	3690	4500	3175	3760	**4486,0**	**1038,7**	**4112,5**
**Drain "fluid sheet"**	675	190	565	740	590	505	260	445	1050	600	**562,0**	**243,0**	**577,5**
**Bleeding**	3700	2600	800	1800	700	650	500	1400	850	520	**1352,0**	**1062,8**	**825**
**"Perspiratio + basal need"**													
**Sum OUT**	10775	8045	5610	6520	5250	7050	4450	6345	5075	4880	**6400,0**	**1888,6**	**5977,5**
**Fluid Balance**	3912	5332	7536	5287	5045	4406	6615	7335	6344	6083	**5789,5**	**1200,4**	**5707,5**

"Fluid sheets" is a paper document for documentation of fluids, diuresis, etc

Take Care (TC) is our electronic record system (manual input, no electronic capture of data)
